# Remote Activation of Enzyme Nanohybrids for Cancer Prodrug Therapy Controlled by Magnetic Heating

**DOI:** 10.1021/acsnano.3c01599

**Published:** 2023-06-26

**Authors:** Beatriz Torres-Herrero, Ilaria Armenia, Maria Alleva, Laura Asín, Sonali Correa, Cecilia Ortiz, Yilian Fernández-Afonso, Lucía Gutiérrez, Jesús M. de la Fuente, Lorena Betancor, Valeria Grazú

**Affiliations:** † Instituto de Nanociencia y Materiales de Aragón (INMA), CSIC-Universidad de Zaragoza, Zaragoza 50009, Spain; ‡ Centro de Investigación Biomédica en Red de Bioingeniería, Biomateriales y Nanomedicina (CIBER-BBN), Madrid 28029, Spain; § Laboratorio de Biotecnología, Universidad ORT Uruguay, Montevideo, 11100, Uruguay; ∥ Departamento de Química Analítica, Universidad de Zaragoza, Zaragoza 50009, Spain

**Keywords:** enzyme prodrug therapy, magnetic nanoparticles, biomimetic silica, nanohybrid, nanoactuation, remote enzyme activation, magnetic heating

## Abstract

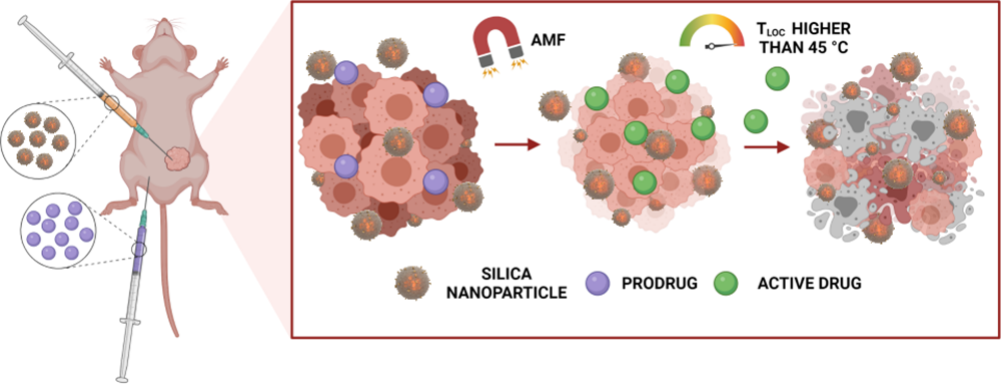

Herein, we have developed nanohybrids (nHs) to remotely activate a therapeutic enzyme for its use in Directed Enzyme Prodrug Therapy (DEPT). The coencapsulation of magnetic nanoparticles (MNPs) with horseradish peroxidase (HRP) using biomimetic silica as an entrapment matrix was optimized to obtain nanosized hybrids (∼150 nm) for remote activation of the therapeutic enzyme. HRP converts indole-3-acetic acid (3IAA) into peroxylated radicals, whereas MNPs respond to alternating magnetic fields (AMFs) becoming local hotspots. The AMF application triggered an increase in the bioconversion rate of HRP matching the activity displayed at the optimal temperature of the nHs (*T*_opt_ = 50 °C) without altering the temperature of the reaction media. This showed that enzyme nanoactuation is possible with MNPs even if they are not covalently bound. After an extensive physicochemical/magnetic characterization, the spatial location of each component of the nH was deciphered, and an insulating role of the silica matrix was suggested as critical for introducing remote control over HRP. *In vitro* assays, using a human pancreatic cancer cell line (MIA PaCa-2), showed that only upon exposure to AMF and in the presence of the prodrug, the enzyme-loaded nHs triggered cell death. Moreover, *in vivo* experiments showed higher reductions in the tumor volume growth in those animals treated with nHs in the presence of 3IAA when exposed to AMF. Thus, this work demonstrates the feasibility of developing a spatiotemporally controlled DEPT strategy to overcome unwanted off-target effects.

## Introduction

Recent investigations on cancer treatment gravitate toward the use of alternative therapies that overcome the current problems of classical ones, *viz*., insufficient concentration of the drug at the tumor site, undesired biodistribution, systemic toxicity, lack of selectivity between cancer and normal cells, and development of resistance.^1^ Enzyme therapy strategies, modeled on the use of enzymes and prodrugs and known as directed enzyme prodrug therapy (DEPT), provide an edge over standard nonspecific therapies due to their potential selectivity. DEPT uses foreign enzymes artificially introduced in the body to convert prodrugs into their active form *in situ* at the desired target. Thus, DEPT strategies could minimize unwanted systemic effects following the direct parenteral administration of the drug.^2,3^ Moreover, DEPT presents an amplifying effect since a single enzyme can activate many prodrug molecules at the tumor site, generating cell death even on adjacent tumor cells (bystander effect) with no need for enzymatic internalization.^4^

Most of the technologies developed for DEPT so far rely on the use of antibodies, lectins, or viruses as enzyme carriers. These carriers face some limitations hampering DEPT clinical application like poor carrier/enzyme/prodrug stability, immunogenicity, rapid clearance of the enzyme, and limited delivery to target areas. However, the success of DEPT depends on a highly selective delivery vehicle for the exogenous enzyme to the tumor site, preventing its recognition by the immune system and its accumulation in healthy tissues.

Enzyme encapsulation emerged as an approach to circumvent some limitations in the application of DEPT. First, it improves the accumulation and retention of the encapsulated therapeutic enzyme within the tumor^5^ by exploiting their well-known enhanced permeability and retention (EPR) effect. EPR provokes the accumulation of nanoparticles in tumors due to a combination of fenestration in the vasculature and poor lymphatic drainage.^6^

Second, immuno-isolation provided by encapsulation aids a possible DEPT treatment by decreasing the reticuloendothelial system (RES) clearance of the enzyme from plasma,^7^ a known cause of ineffective enzyme concentrations at the tumor site. Besides, it could also help to reduce the induction of neutralizing antibodies due to repeated administration of the therapy, which impairs enzyme activity.^8,9^ Finally, enzyme encapsulation improves proteolytic stability, preserving the enzyme activity *in vivo* and generating nanocomposites with high volumetric enzyme activity.^10^ It also provides a cover shell that serves as a surface for anchoring active targeting agents that could facilitate the interaction with target cells (e.g., glucose, folic acid, antibodies).^11^

Although encapsulating enzymes for DEPT offers several benefits, problems related to selectivity persist and present a significant challenge in nanotherapeutics.^12^ For instance, the accumulation of nanoparticles in the liver and spleen is a major concern, as the biological role of these organs relies on fenestration in their vasculature to eliminate such materials, akin to the EPR effect.^13^ Furthermore, the current use of mesophilic enzymes which are active at body temperature implies that off-target effects are unavoidable. Besides, the use of enzymes with optimal temperatures higher than body temperature is yet to be investigated. Therefore, the implementation of strategies that could provide spatio-temporal control over the activity of therapeutic enzymes with optimal temperatures higher than 37 °C through external stimuli could surpass the current off-target activation paradigm in DEPT.^14^

It is well-known that inorganic nanoparticles (NPs) can be activated as hotspots by light or alternating magnetic fields (AMF) as extrinsic energy sources and, thus, act as nanoactuators.^15^ In contrast to classical magnetic hyperthermia, which aims to induce cell death through a global temperature increment using magnetic heating, local magnetic hyperthermia relies on a temperature rise in the vicinity of a magnetic nanoparticle (MNP) without a macroscopic temperature increase.^16−19^ So far, this local heating effect has been mainly explored in therapy to trigger direct cell ablation for cancer treatment or provide spatio-temporal control of a drug^20^ or enzyme release from nanotherapeutics.^21^ In one of the few examples found in the literature, a light-responsive nanostructure was used for triggering the local thermal activation of glucose oxidase to achieve spatio-temporal control of enzyme-based starvation therapy.^22^ In another example, iron oxide magnetic nanoparticles (MNPs) exposed to AMF were utilized as nanoactuators to trigger the expression of the enzyme cytosine deaminase able to convert the cytotoxic 5-fluorouracil from the prodrug 5-fluorocytosine.^23^ Finally, the use of MNPs to achieve the heat-triggered control of a glucose oxidase (GOx)–peroxidase nanozyme cascade reaction was recently reported for the production of intracellular reactive oxygen species (ROS).^24^ Therefore, the remote activation of NPs as local heating sources to enhance the catalytic activity of therapeutic enzymes remains practically unexplored.

In the aforementioned examples, the direct binding of the enzyme to the nanoactuator is what enables the remote control of its activity. However, there is still a knowledge gap in the ability of MNPs as nanoactuators when enzymes are not directly attached to these nanoparticles.

**Scheme 1:**
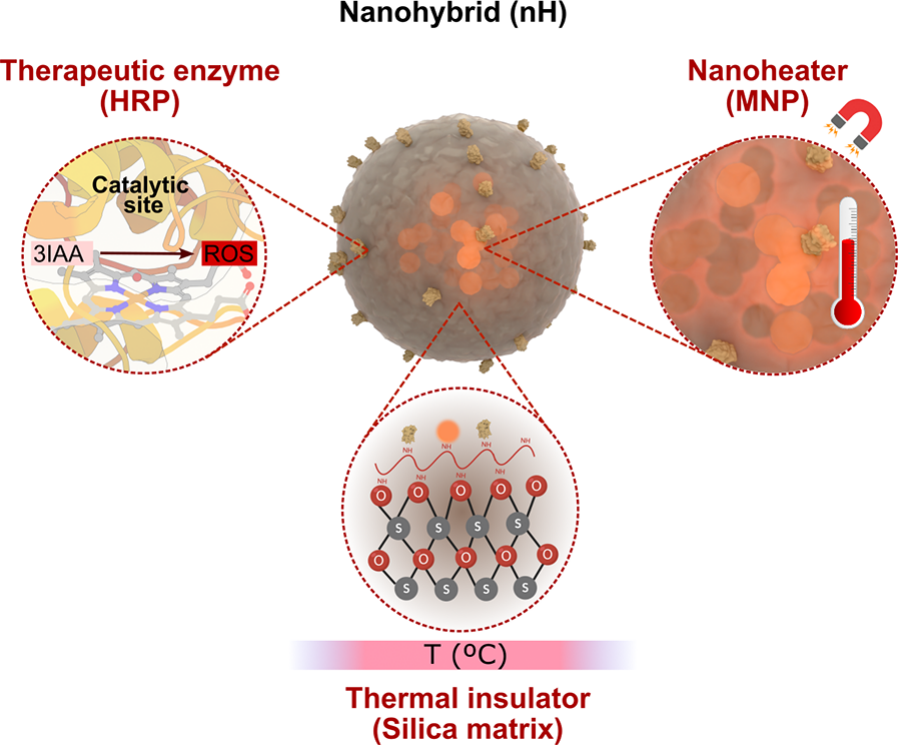
Schematic Illustration of the Three Main Components of the Developed nHs Modeling of the nHs was prepared by Blender and 3D Protein Imaging Studio.^25^ 3D structure of HRP (PDB entry 1HCH) was rendered with 3D Protein Imaging.^25^.

Thus, herein we developed a hybrid nanoparticle that ensures the coencapsulation of a prodrug converting enzyme and MNPs, allowing remote control of prodrug conversion. As illustrated in Scheme 1, this hybrid nanosystem contains three components: horseradish peroxidase (HRP) as a therapeutic enzyme, magnetic nanoparticles as AMF-induced local heaters, and biomimetic silica as the container.

HRP was selected as a therapeutic enzyme because of its ability to convert indole-3-acetic acid (3IAA), a vegetable nontoxic hormone, into peroxylated radicals with widespread cytotoxic activity in tumor cell lines.^26^ Moreover, HRP was a suitable choice to test its remote thermal activation as it has an optimum temperature in the range of 45–50 °C^27−30^ and, therefore, a lower biocatalytic activity at body temperature. MNPs were selected as nanoactuators for their activation into hotspots using AMF as it has a higher penetration into soft tissues compared to light as an external energy source.^31,32^ Moreover, MNPs have the added advantage over plasmonic nanoparticles of being biodegradable and present a much finer modulation of the remotely triggered heating power output.^33^ Silica was selected as an encapsulating matrix not only due to its suitability for *in vivo* applications owing to its enzyme stabilization properties, biodegradability, biocompatibility, and low toxicity,^34,35^ but also due to its well-known thermal insulating capacity.^36^ In fact, it has already been shown that the interior of mesoporous silica nanoparticles that encapsulated MNPs becomes much hotter than the macroscopic solution and cools to the temperature of the latter in a matter of seconds after the AMF is turned off.^36^ These previously published results led us to hypothesize that even if not directly attached to the MNPs, the activity of therapeutic enzymes could become switchable due to their silica coencapsulation with nanoheaters. Moreover, an entrapment strategy for integrating enzymes within the Si matrix offers advantages in terms of increased structural freedom of the immobilized enzymes, as compared to their chemical attachment to a rigid surface. The three-dimensional crosstalk between enzyme and material in an encapsulation method facilitates a more intense interaction that may lead to enhanced enzyme stabilization.^37^ Moreover, our proposed encapsulating strategy may avoid the problems that arise from unfavored orientations of enzymes bound directly to MNPs. This last approach has faced challenges in terms of substrate accessibility and the extent of enzyme remote activation.^38−40^ Consequently, an encapsulation method may provide versatility and even expand the range of therapeutic enzymes that can be remotely activated.

A biosilification approach was selected for the coentrapment of MNPs and HRP molecules in biomimetic silica. This occurs while the silica matrix is being formed *via* catalysis by a polyamine molecule in the presence of silicic acid.^41^ We have previously shown that the use of this biomimetic biomineralization strategy allows the entrapment of HRP and MNPs forming hybrid nanoparticles (∼500–600 nm mean diameter) without significantly altering HRP biocatalytic properties while increasing its thermal stability.^37^ Herein, we have optimized the synthesis to obtain smaller hybrids (150 nm mean diameter), more suitable for therapeutic use, in which the integration of HRP molecules and MNPs is demonstrated. Besides, this work demonstrates that the entrapped enzyme could be remotely activated even if it is not directly bound to the surface of the AMF-responsive nanoheaters. This ability to remotely control HRP activity by magnetic heating was used to increase its prodrug conversion and cytotoxicity effect on pancreatic carcinoma cells (MIA PaCa-2) when AMF was applied *in vitro.* Besides, *in vivo* experiments using MIA PaCa-2 derived xenograft models also confirmed the higher therapeutic efficiency of nHs when 3IAA bioconversion was sped up by AMF application.

Although recent strategies have successfully demonstrated the tumor-toxic effects of nanoenzymes with redox 3IAA conversion activity resembling HRP,^42^ our findings represent evidence of remote spatio-temporal control over the prodrug converting activity of HRP. Moreover, the developed coentrapment strategy avoids the requirement for enzyme binding to the nanoactuator for activity regulation, eliminating the necessity for *ad hoc* biofunctionalization strategies. Therefore, this approach provides versatility for the coentrapment of other therapeutic enzymes, thereby expanding the concept of prodrug activation beyond redox reactions.

## Results and Discussion

### Synthesis and Physicochemical Characterization of the Nanohybrids (nHs)

**Figure 1:**
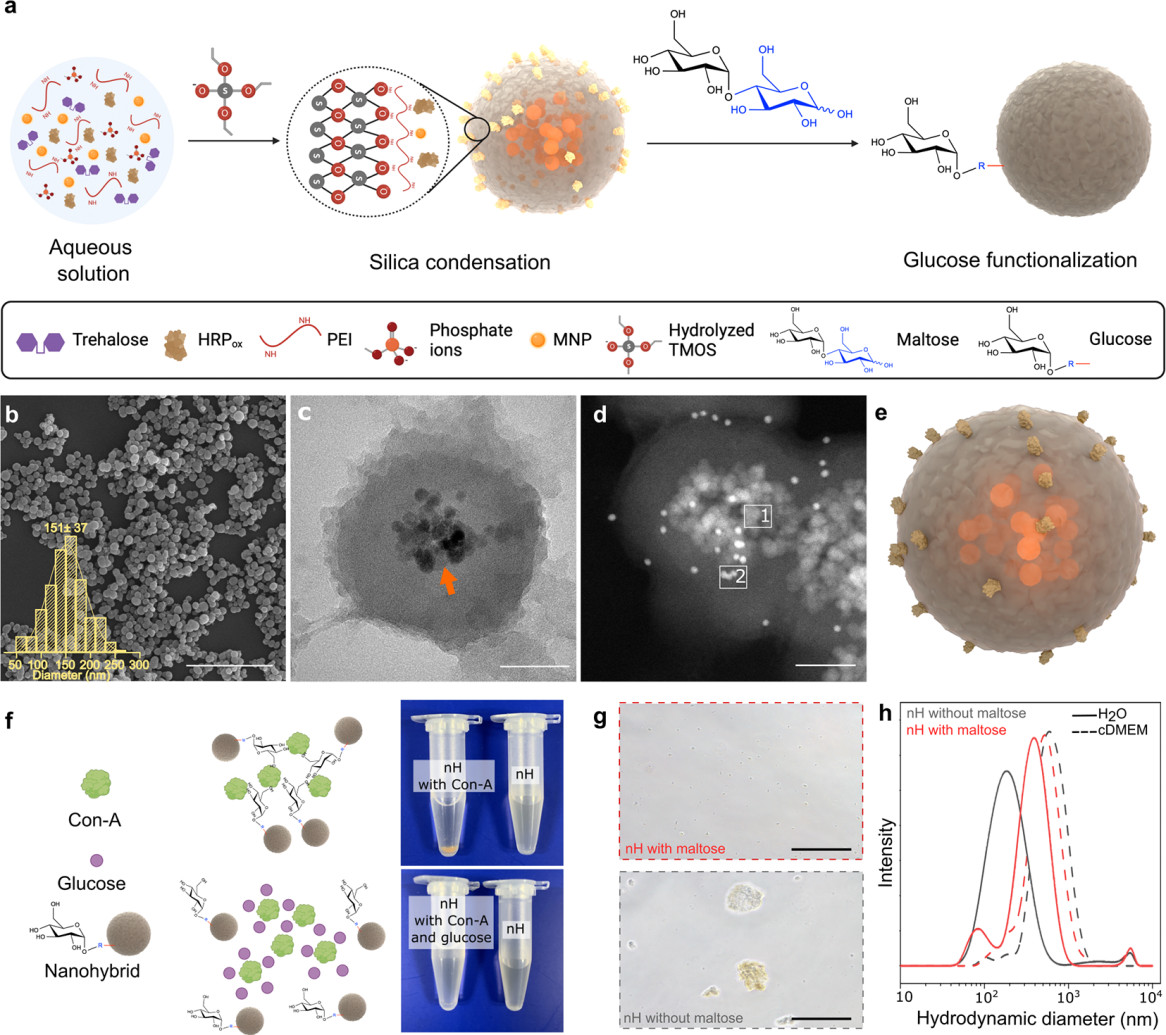
Synthesis and structural characterization of enzyme-MNPs loaded nHs. a, Schematic illustration of the synthesis process. “R” in blue refers to the linearized glucose unit while the red line indicates its secondary amine bond with the exposed primary amines of the nH. Image created with Biorender. b, SEM micrograph with histogram and log-normal fitting curve of the size distribution. Scale bar: 2 μm; (*n* = 120). c, TEM micrograph of a section of nHs. The orange arrow points to the MNPs core within the silica matrix. Scale bar: 50 nm. d, STEM-HAAD of a section of nHs. Scale bar: 50 nm. Rectangles 1 and 2 correspond to the MNPs and gold (Au) areas respectively analyzed by EDX: Further images can be seen in Figure S4. e, Modeling of the nHs render with 3D Protein Imager and Blender.^25^ f, (left) Schematic representation of Con-A aggregation studies to determine the bioactivity of glucose residues introduced on the nHs surface. (right, upper) Aggregation of maltose-functionalized nHs due to the presence of Con-A. (right, lower) Aggregation disappeared as free glucose is added due to its ability to compete for the active sites of Con-A with the glucose residues introduced at the nH surface. g, Optical micrograph of the nHs with (upper, red) and without (lower, gray) maltose in cDMEM. Scale bar: 50 μm. h, DLS analysis of nHs functionalized (red) or not (gray) with maltose in water (solid line) and cDMEM (dashed line).

AMF-responsive nHs were generated via coencapsulation of the therapeutic enzyme (HRP) and MNPs (Figure S1 and Table S1) in a biomimetic silica matrix with a fast reaction that is performed in minutes under mild enzyme-compatible conditions (Figure 1a). Silica deposition was achieved by hydrolyzed tetramethyl orthosilicate (TMOS) addition and driven by phosphate ions that assist the assembly of the acid–base catalyst polyethyleneimine (PEI) in a network entrapping HRP molecules and MNPs present in the reaction mixture. Key factors for nH synthesis turned out to be (i) the previous oxidation of HRP (Figure S2a and Figure S3a), which allows the formation of Schiff bases between the generated aldehyde groups at the enzyme’s sugar chains and the amine groups of the PEI, ensuring the rigidification and integration of the enzyme 3D structure; (ii) a low concentration of phosphate ions (5 mM) which ensures the generation of nanosized hybrids (Figure S3b); and (iii) a PEI of 1300 Da from the two different molecular weight PEIs tested (1300 and 60 000 Da) (Table S3).

From all the coencapsulation conditions tested (Table S2 and Table S3), condition 3 was chosen not only due to high immobilization (68 ± 3%) and expressed activity percentages (60 ± 7%) of the entrapped HRP obtained but also due to the reduced size of the nHs obtained, which favors the integration of the MNPs within the silica matrix (Figure S3b). Indeed, a high iron entrapment efficiency (90 ± 10%) was achieved in this condition, corresponding to ∼6 × 10^12^ MNP/mg silica (Table S4a,b). Scanning electron microscopy (SEM) images showed that the obtained nHs were sphere-shaped nanoparticles with an average particle size of 151 ± 37 nm (Figure 1b). The entrapment of the MNPs was verified by transmission electron microscopy (TEM) as they were observed to be homogeneously distributed in the core of the nHs (Figure 1c).

The distribution of the HRP molecules within the silica matrix that surrounds the magnetic core of the nHs was demonstrated by immunogold labeling and High Angle Annular Dark Field Scanning Transmission Electron Microscopy (HAADF-STEM) analysis of ultrathin sections of a gelatin-based resin where the hybrids were previously embedded (Figure 1d and Figure S4). The elemental mapping of these sections based on energy-dispersive X-ray spectroscopy (EDX) confirmed a core–shell structure (Figure 1e and Figure S4e). Further insight into the integration of the enzyme molecules within the nH structure was obtained by FTIR characterization. The presence of amide bonds indicates the covalent interaction between the aldehyde groups introduced within the polysaccharide chains of HRP and the amino groups of the PEI confirming the formation of a 3D-covalent matrix (Figure S5a). The combined insight into the architecture of the hybrids provided by these experiments may explain the activity reduction of the enzyme upon immobilization. For those molecules within the matrix, partition problems of substrates and products might be expected. Furthermore, for those on the surface, the active site might be occluded restricting their access to substrates and diminishing the apparent activity observed for the nHs. However, as usually occurs for other immobilized enzymes, the advantages provided by the matrix integration may counterbalance the activity loss.

The organic/inorganic (O/I) composition of the hybrids was also confirmed by thermal gravimetric analysis (TGA) revealing a 30/70 O/I wt% ratio (Figure S5b). Besides, standard N_2_ adsorption assays at 77 K (Figure S5c) showed that the obtained hybrid possesses a specific BET surface area of 21 m^2^/g. According to the t-plot, 10 m^2^/g corresponds to the micropore area and 11 m^2^/g to the external surface area. These results agree well with previous values reported for silica NPs obtained by Stöber synthesis method,^43^ indicating that the obtained nHs have similar textural properties.

The nHs obtained were found to aggregate when exposed to complex media. To overcome this issue, we used a functionalization approach with maltose (Glc α1–4 Glc) through reductive amination reaction^44^ (Figure S2b). This process entails the covalent bonding of the aldehyde group of the terminal reducing glucose (Glc) unit of maltose with the amine groups present on the nHs, leading to the exposure of the remaining functional glucose unit on the surface of the nHs. To confirm the functionalization, affinity-based aggregation studies were performed by adding concanavalin A (Con-A) lectin as it has multiple selective binding sites to glucose. As could be observed in Figure 1h, Con-A binding caused nH precipitation, proving an interaction with glucose moieties at the nH surface. The specificity of this interaction was probed by the subsequent addition of an excess of soluble glucose, which acts as a competing inhibitor for the union to Con-A, which resulted in the dissociation of the Con A-triggered nH aggregates.

The resulting nHs proved to have enhanced colloidal stability as neither visible aggregates nor a change in the hydrodynamic diameter were observed when the functionalized nHs were incubated in complex biological media (Figure 1g,h).

### Characterization of nH Heating Efficiency and Magnetic Properties

**Figure 2:**
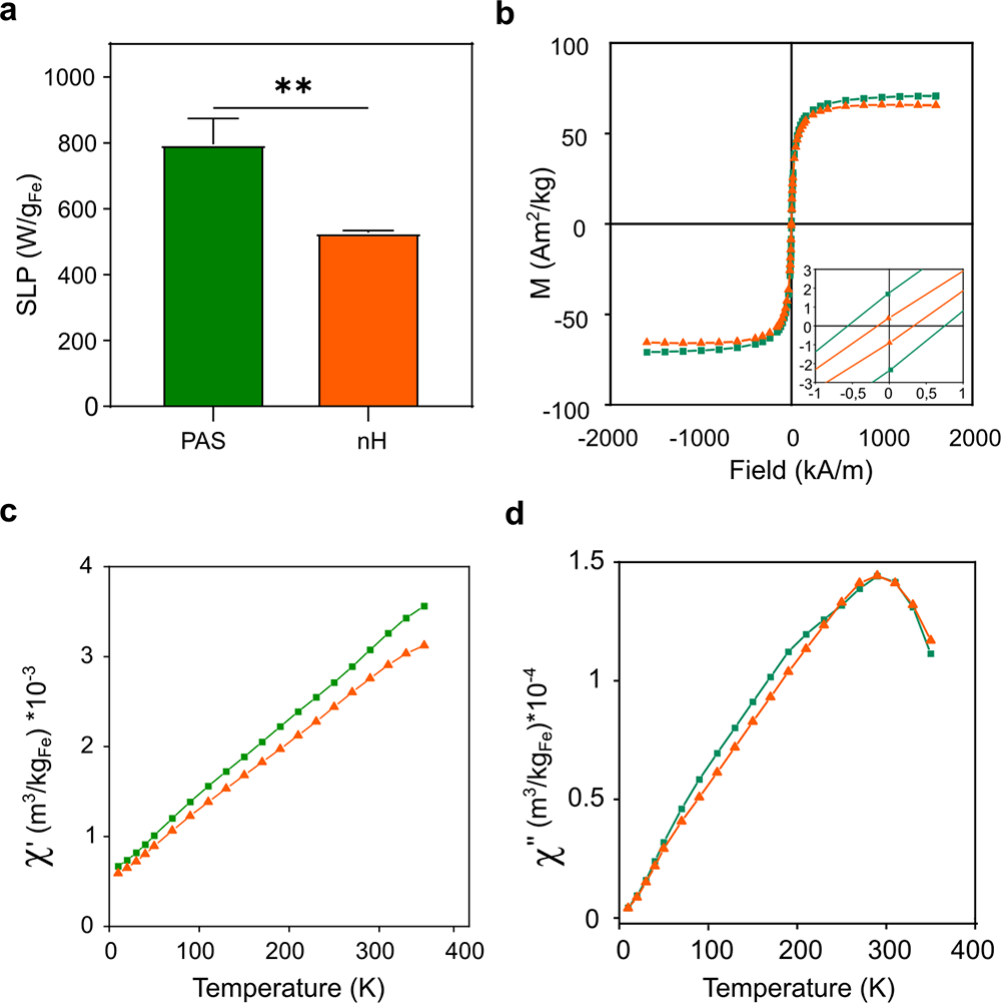
Heating efficiency and magnetic characterization of nHs. a, SLP registered for bare MNPs (green) and nHs (orange) (0.9 Fe mg/mL) at 763 kHz and 36 mT. The bar graph represents mean ± sd, *n* = 3. Unpaired T-test (**p < 0.01). b, Magnetization-field hysteresis curves of bare MNPs (green square) and nHs (orange triangle) at 300 K. (inset) Zoom of the central part of the cycles. c,d, Temperature dependence of the AC magnetic susceptibility (c, in-phase component, and d, out-of-phase component) of bare MNPs (green square) and nHs (orange triangle).

The specific loss power (SLP) is defined as the power transformed into heat per mass of nanoparticles. This is a way to compare the magnetic heating efficiency of different systems and in our case of bare and coentrapped MNPs within the silica matrix of the nHs. The measurements obtained clearly showed that the entrapped MNPs presented a significant reduction in their SLP value (795 ± 77 W/g_Fe2O3_ for bare MNPs and 527 ± 7 W/g_Fe2O3_ for nHs) (Figure 2a and Figure S6). Owing to the way SLP is determined, results relate to MNPs capacity to increase the global temperature of the media in which nHs are suspended (macroscopic heating), but it fails to measure the heat gradient generated in close vicinity of the MNPs (local heating) upon field exposure. Several reasons could explain the decrease in the SLP values observed. On one hand, if MNP aggregation occurs during nHs preparation, their magnetic properties as well as their heating properties may be affected.^45,46^ On the other hand, this difference in SLP values could also be related to the well-known insulating properties of silica,^47−50^ which could cause a decrease in the heat transference from well-functioning nanoheaters to the aqueous media. While this property could be seen as a drawback for traditional magnetic hyperthermia (MHT) applications, it would be beneficial for the remote tuning of the activity of coentrapped enzymes that are not directly linked to the surface of the MNPs.

A thorough magnetic characterization was performed to elucidate the cause of the observed SLP value reduction. First, the magnetic hysteresis curves of the nHs were recorded at room temperature (300 K). In these measurements, the sample magnetization (M) was measured as a function of the applied field (H). This type of measurement informs possible transformations of the MNPs during the encapsulation process, showing that the entrapped MNPs maintained their superparamagnetic behavior as well as their saturation magnetization value (*M*_s_) at this temperature (Figure 2b and Figure S7a). Indeed, the *M*_s_ value of both the entrapped MNPs and the bare ones was very similar, ∼70 Am^2^/kg Fe_2_O_3_, in agreement with the typical values of maghemite (76 Am^2^/kg Fe_2_O_3_).^51^ A different technique, the measurement of the temperature dependence of the AC magnetic susceptibility, was used to assess the aggregation of the particles within the nHs. For a given type of nanoparticles, a shift in the temperature location of the susceptibility maxima informs about different degrees of dipolar interactions, which is related to the aggregation of the particles. In this case, the nH synthesis did not generate MNPs aggregation, as bare and entrapped MNPs showed similar in-phase (χ′(*T*)) and out-of-phase (χ″(*T*)) susceptibility behaviors, with the maxima of the out-of-phase susceptibility located at the same temperature (∼290 K) for both samples (Figure 2c,d and Figure S7b). Thus, these results support the hypothesis of the silica matrix acting as a thermal insulator, that reduces the heat dissipation into the surrounding media and maintains the heat released from the particles within the nHs for longer times.

### Biochemical Characterization and AMF-Triggered Enzyme Activity

**Figure 3:**
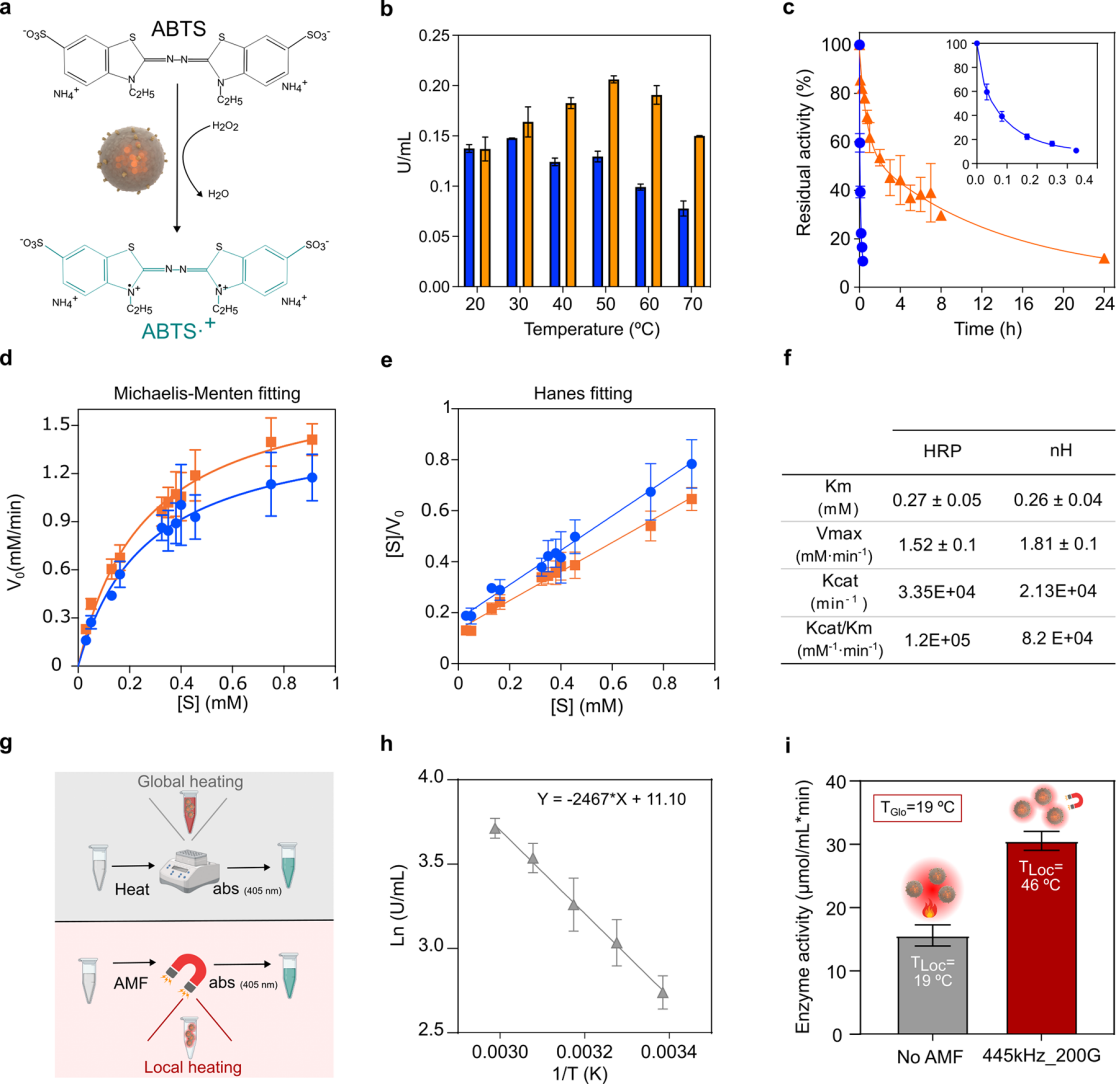
Biochemical properties of nHs and AMF-triggered entrapped-HRP activation. Blue circle, soluble HRP. Orange triangle, nHs. a, Schematic conversion of the substrate ABTS by HRP. b, Temperature profile of enzyme activity. The U/mL values of the enzyme preparations were normalized at 20 °C. c, Thermal stability at 50 °C. Relative activity is expressed as the percentage of the activity at *t*_0_ for the soluble and entrapped enzymes. d, Michaelis–Menten nonlinear fitting plot and e, Hanes linear fitting for determination of kinetic parameters. f, Kinetic parameters of HRP and nHs. g, Schematic representation of the approach to infer the local working temperature of the entrapped HRP under AMF application. h, Arrhenius plot of the nHs. i, Activity of the nHs after 5 min at 19 °C or upon 5 min exposure to a frequency (*f*) of 445 kHz and a field (*H*) of 200 G while the global temperature in the reaction media was 19 °C. Estimated local temperature by interpolation in the Arrhenius plot under AMF conditions is indicated as *T*_Loc_ is local temperature and *T*_Glo_ is global temperature of the reaction media.

We have further characterized the nHs by studying the effect of the temperature on the enzymatic activity using a colorimetric assay with ABTS as substrate (Figure 3a). The oxidized soluble enzyme at the incubation conditions tested shows an optimal temperature range of 20–30 °C, while that of the entrapped one shifted to 50 °C (Figure 3b). This behavior is often observed in immobilized enzymes,^52−55^ as the immobilization process may provoke slight changes in the biocatalyst structure leading to shifts in optimal parameters or stabilization of the 3D structure that impact even short time activity assays. Indeed, HRP entrapment provided its stabilization (120×) with the half-life time at 50 °C of 4 h for nHs compared to 2 min for the soluble enzyme (Figure 3c). Biocatalyst inactivation was modeled based on a two-stage series inactivation mechanism with *k*_1_ and *k*_2_ being 52 and 125 times lower, respectively, than those of soluble HRP. These results suggested that the encapsulation within the silica matrix leads to a higher 3D stability delaying its complete denaturation. The thermal stabilization achieved is of great importance in the context of this work in which magnetic heating is the remote stimulus selected for tuning the enzyme therapeutic activity.

Kinetics analyses (Figure 3d,e,f) were performed to study if HRP reaction rates are affected due to its encapsulation. The apparent *K*_M_ obtained for the nHs for ABTS oxidation was similar to the *K*_M_ of the soluble oxidized enzyme (0.26 ± 0.04 and 0.27 ± 0.05 mM, respectively). This indicated no partition problems of substrates and products or significant modifications in the active site upon its encapsulation. Besides, *V*_max_ values for the soluble HRP and nHs were 1.52 ± 0.1 and 1.81 ± 0.1 mM/min, respectively, whereas their *K*_cat_ values were 3.3 × 10^4^ and 2.13 × 10^4^ min^–1^, indicating a slight impact of entrapment on its bioconversion activity providing a catalytic efficiency of 1.2 × 10^5^ min^–1^ mM^–1^ and 8.2 × 10^4^ min^–1^mM^–1^, respectively.

We have already shown that the constituents of the nHs preserved their desired properties for achieving remote thermal activation of the prodrug conversion. As the focus of this nanoactuation strategy is based on the well-known impact of the temperature on enzyme activity, we studied the effect of AMF application on the ABTS oxidation rate (Figure 3g).

The activity of the soluble and entrapped oxidized enzyme vs temperature profile (below the inactivation temperature) was used to obtain the linearization plot of the Arrhenius equation to directly correlate temperature with initial rates of ABTS oxidation (Figure 3h and Figure S8). Therefore, the correlation allowed us to infer that upon application of an AMF of 20 mT of amplitude and a frequency of 445 kHz during 5 min, a significant increase in the ABTS oxidation rate of the entrapped HRP was triggered compared to its bioconversion without AMF exposure (Figure S8c). Our findings proved that even though the global media temperature remained unaltered at 19 °C during AMF application, the activity of the nHs exposed to the magnetic field, corresponded to a reaction temperature of 46 ± 2 °C as inferred by linear regression fit (*R*^2^ = 0.997) in the Arrhenius plot (Figure 3h,i). The stability of the enzyme upon AMF application was checked without detecting any significant reduction in its activity (Figure S9).

It has to be taken into account that the temperature on the surface of the nanoparticles is likely different from the local temperature sensed by the enzyme, both considering the architecture of our nHs and the fact that the enzyme is not directly attached to the MNPs. Thus, a deeper understanding of the heat transfer inside the nHs might provide information for future alternate designs (*e.g*., enzymes with different optimal *T*, MNPs with different heating capacities, and thermally responsive Si). Indeed, advancements in measuring the temperature of the nanoheater currently follow two strategies: (i) utilizing a second nanoparticle for thermometry (known as the dual-particle approach) and (ii) attaching a molecular thermometric probe to the surface or outer shell of the nanoheater (known as the single-particle approach).^36,56−60^ However, the use of any of those methods implies the modification of the nH surface or the addition of a new component into the silica matrix. This may result in a different hierarchical integration of the components that may alter the nH properties, especially the efficiency of the remote activation of the HRP. Thus, our chosen methodology is in accordance with our primary goal of identifying the specific local temperature that directly impacts the rate of the enzymatic reaction being studied.

Our results demonstrate the successful remote thermal activation of the therapeutic enzyme, which was coentrapped with MNPs within a silica matrix but not directly attached to them. This activation occurs locally through the application of an external AMF without elevating the overall temperature of the reaction medium or compromising the stability of the enzyme.

### Remote On/Off Switching of *In Vitro* Enzyme Prodrug Activation

Once the AMF-tunability of the entrapped HRP activity was demonstrated, the feasibility to trigger prodrug (3IAA) bioconversion remotely in biological environments was examined. 3IAA is a plant auxin with very low toxicity even when administered in high doses (*e.g*., >100 mg/kg body weight), but its oxidation dramatically enhances the killing of mammalian cells. Indeed, 3IAA undergoes one-electron oxidation in the presence of HRP to form carbon-centered free radicals such as indol-3-yl, skatolyl, 3-methylene-2-oxindole, and oxindol-3-yl radicals. These biologically reactive products induce oxidative degradation (DNA, lipid, and protein oxidation), resulting in cellular cytotoxicity and apoptosis of target cells.^61^ The application of previously reported HRP-3IAA systems has shown antitumor activity in the treatment of both *in vitro* and *in vivo* models of a wide variety of tumors.^26,62−64^ Besides, the use of HRP has several advantages including that it does not require hydrogen peroxide for oxidation of 3IAA, exhibits optimal catalytic activity at the characteristic low pH (6.0–6.5) of tumor microenvironments, and presents activity under high and also low oxygen tensions (hypoxic regions of tumors).^61,65^

To demonstrate the AMF-tunable therapeutic potential of the developed nanoplatform, the inherent cytotoxicity of the obtained nHs was first assessed in the human pancreatic carcinoma cell line (MIA PaCa-2). In the absence of the prodrug, high cell viability was observed (>80%) even after 24 h of incubation with nHs concentrations up to 250 μg mL^–1^ (Figure S10a).

**Figure 4:**
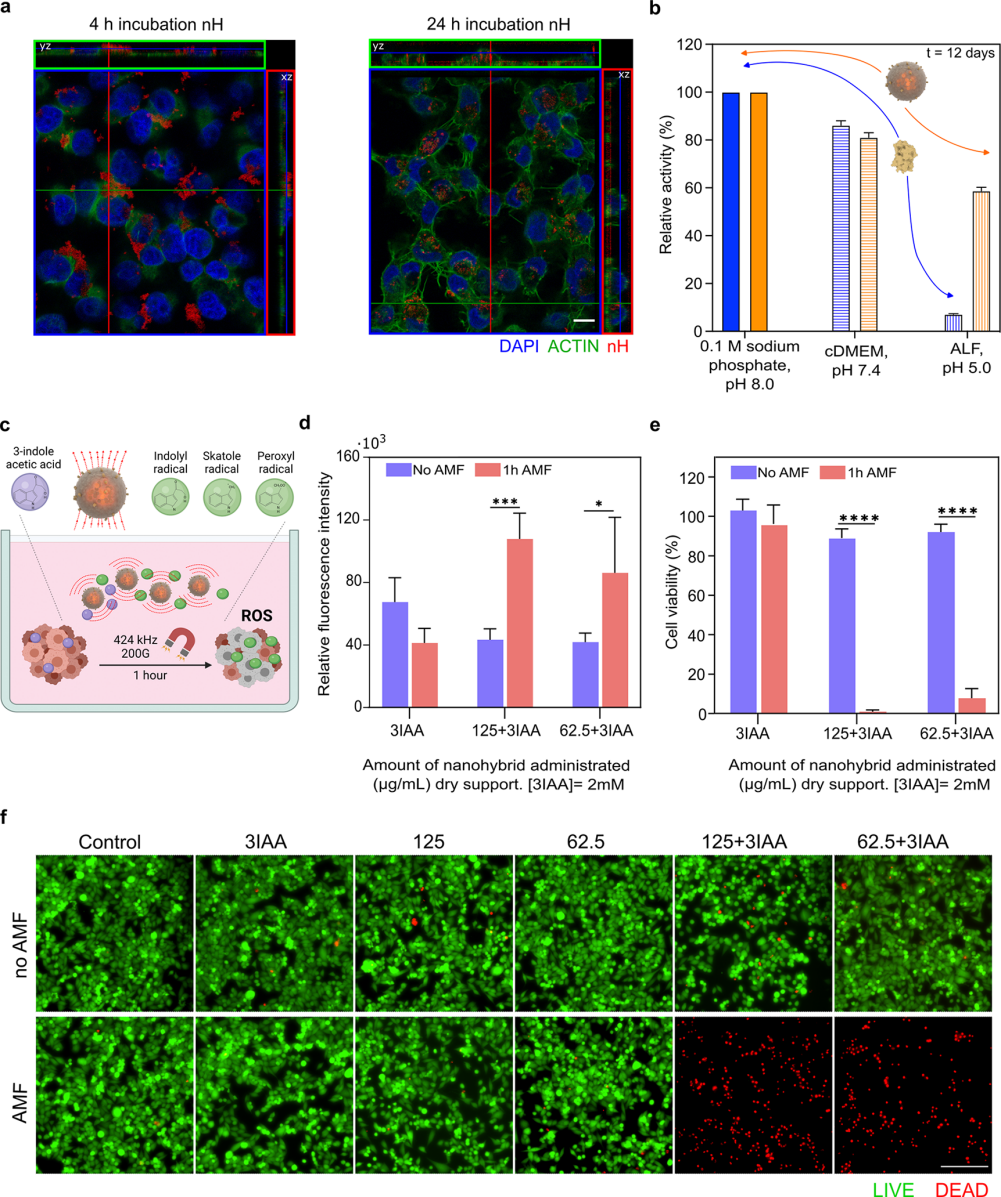
AMF-triggered prodrug conversion using nHs. a, Orthogonal projections of a confocal section of nHs (200 μg mL^–1^) incubated with MIA PaCa-2 cells for 4 h (left) or 24 h (right). The nucleus is stained with DAPI (blue) and actin by Phalloidin-488 (green). nHs were detected by iron self-reflection signal (artificially colored in red). The scale bar is 10 μm. b, Protective effect of the silica matrix over the HRP activity. The relative activity of the soluble (blue) and entrapped (orange) HRP at 0.1 sodium phosphate pH 8.0 (solid fill), cDMEM (horizontal lines), and artificial lysosomal fluid (ALF) (vertical lines) at day 12 is shown. Further data can be seen in Figure S11. c, Scheme showing conversion of 3IAA into peroxylated radicals with tumor cytotoxic activity. d, Effect of the AMF-triggered drug conversion on intracellular ROS levels measured after 6 h of the application of AMF using DCFH-DA probe (red bars). The same incubation conditions but without AMF application were used as a control (purple bars). e, Cell viability determined by MTT after 24 h of AMF application (red bars). The same incubation conditions but without AMF application were used as a control (purple bars). f, Live/dead cell viability assays using calcein AM and EthD-1 analyzed after 24 h of AMF application by fluorescence microscopy. Live cells are labeled in green (calcein AM labeling) and dead cells in red (EthD-1 labeling). The scale bar is 200 μm. Common aspects for the experiments described in c, d, and e were as follows: (i) when AMF was applied, cells were exposed to an AMF of 424 kHz 20 mT during 1 h; (ii) when incubated with nHs, two different nHs concentrations were used (62.5 and 125 μg mL^–1^); (iii) when the prodrug was applied, cells were exposed to 2 mM of the prodrug 3IAA that was added alone or together with the different nHs concentrations, respectively. Different controls were carried out, all of them exposed or not to AMF: cells without any treatment, cells incubated only with the prodrug (2 mM), and cells incubated only with the different concentrations of nHs. Histogram represents mean ± SEM, *n* = 3. Two-way ANOVA, followed by Tukey’s multiple comparisons test (*p < 0.05; **p < 0.01; ****p < 0.0001).

As previously indicated, the functionalization process employed to enhance the colloidal stability of the nHs resulted in the exposure of functional glucose units on their surface, which may favor nH–cell interaction. Indeed, glucose functionalization can confer bioactivity and targeted delivery properties to NPs by exploiting the upregulation of glucose transporters in tumor cells. This well-recognized hallmark is attributed to the high glucose consumption exhibited by these cells.^66−69^ In fact, nH–cell interaction was confirmed by confocal microscopy after incubation for up to 24 h with MIA PaCa-2 cells (Figure 4a, Figure S10b). Although endocytosis of the nHs may not be necessary to trigger cell death, as the toxic oxidative species produced upon 3IAA bioconversion have a robust bystander effect, our results suggested that a possible internalization of the nHs may occur, mediated by glucose receptors expressed at the cell surface.

Considering the observed internalization of the nH after 24 h, we investigated the stability of the nHs in complex biological media over relevant time periods. The stability of each counterpart of the nH was studied separately, since the instability of one of them could impact the entire nanosystem. Our findings indicate that incubation in complex media did not alter the catalytic activity of either the soluble or entrapped forms of the HRP, nor did it cause changes in the size of the silica nanoparticles compared to the control after 23 days of incubation (Figure 4b and Figure S11b,c). As our results support the internalization of the nHs, we, therefore, analyzed the stability in artificial lysosomal fluid (ALF) to mimic the acidic intracellular environment (pH 5.0) and the presence of numerous proteases. We observed no degradation of the carrier (silica matrix) or the nanoactuator (MNPs) after 23 days of incubation. Moreover, the entrapped HRP showed increased stability against proteinase degradation and pH denaturation compared to its soluble form (*t*_1/2_ of 1 h for the soluble enzyme, *t*_1/2_ of 10 days for the nHs, ∼249-fold stabilization factor), confirming the protective role of the silica shell (Figure 4b and Figure S11b,c,d).

To demonstrate the remote activation of prodrug conversion by magnetic heating, cells were incubated with the nHs (62.5 and 125 μg mL^–1^) (equivalent to 3.2 ± 0.1 and 6.5 ± 0.3 HRP mIU, respectively) and 2 mM of 3IAA. Conditions for AMF application were selected considering the biological limit for the product field (*H*) × frequency (*f*) of the AMF applied. Atkinson and Brezovich defined the limit at 4.85 × 10^8^ A m^–1^ s^–1^, while Hergt and Durtz estimated this limit up to 5 × 10^9^ A m^–1^ s^–1^. However, new safety values have been defined up to 9.59 × 10^9^ A m^–1^ s^–1^.^15,70^ Considering this, the condition selected for this study (1 h of *f* 424 kHz and H 20 mT; *H* × *f* = 6.7 × 10^9^ A m^–1^ s^–1^) meets the biological safety limits to treat cancer in living organisms (Figure 4c).

After AMF application, the cells were further incubated at 37 °C in an incubator supplied with 5% CO_2_ until different bioassays were used for a holistic assessment of the cell biological responses elicited by the treatment. Thus, intracellular ROS levels were assessed after AMF exposure since it has been reported that not only biological active radicals (*e.g.*, indolyl, skatolyl, and peroxyl radicals) but also oxygen reactive species (ROS, *e.g*., O^2–^, and H_2_O_2_) are released upon the prodrug oxidation.^71^ The cell-permeant fluorogenic dye 2′-7′ dichlorofluorescein diacetate (DCFH-DA) was used to quantitively detect the induction of ROS, as it is deacetylated inside the cell by cellular esterases to a nonfluorescent compound, the presence of ROS triggered its oxidation into highly fluorescent 2′-7′dichlorofluorescein (DCF). As depicted in Figure 4d, ROS levels were significantly increased when AMF was applied to cells incubated with nHs and the prodrug. The control experiments revealed that neither the nH itself nor 3IAA itself nor their separate exposure to AMF has any significant contribution to ROS generation (Figure S12a). This strongly indicates that AMF application triggers an increase in the nH rate of 3IAA bioconversion into toxic oxidative species. This was confirmed by performing an MTT assay, as it is one of the most used colorimetric assays to assess cytotoxicity/cell viability by reflecting the total metabolic activity of a cell population.^72^ In fact, compared to the 10% cell death observed when AMF was not applied (Figure 4e and Figure S12b), a reduction of more than 90% was triggered when AMF was applied in the presence of both nHs and the prodrug.

The results obtained were also confirmed by Live/Dead assay, which provides a simultaneous indication of the biochemical (esterase activity) and physical properties (membrane integrity) of the cells. As depicted in Figure 4f, the observation by fluorescence microscopy of cell samples treated with nHs/3IAA demonstrated a complete loss of cell viability only in the presence of AMF. When AMF is not applied to cell samples also treated with the combination of nHs and 3IAA, most observed cells are alive having morphology and spreading like nontreated ones (control cells). Moreover, there was no temperature alteration of the cell culture media during AMF application (Figure S12c) nor cell death even when applying AMF to nHs without prodrug (Figure 4f and Figure S12b), demonstrating that the observed cell death is not an effect of the direct magnetic heating of the cells but due to the selective activation of prodrug conversion. This is consistent with the nH architecture, in which the silica shell serves not only as a thermal insulator but also as a barrier to prevent the direct effects of MNP localized heating on cellular components.

### Metabolization of the nHs *In Vivo*

Based on the observed improved stability of the nH *in vitro* (Figure 4b and Figure S11), we rationalized that the *in vivo* therapy application could follow a single nH intratumoral injection (i.t.) and several consecutive applications of the prodrug together with AMF. This therapeutical scheme would be successful provided that the nHs mostly remain in the tumor upon injection.^73^ For that reason, we have analyzed the remaining nH concentration in the tumor tissue together with their biodistribution over time among other organs after i.t. Using the MIA PaCa-2 derived xenograft mouse model and magnetic measurements, the liver, spleen, and tumor tissues were analyzed. The temperature dependence of the AC magnetic susceptibility for the previous organs was measured and compared with that of the injected particles (Figure S13a). Our results indicated that the nHs remained in the tumor, as no remaining magnetic particles were detected in the spleen or in the liver. In fact, when quantifying the number of nHs in whole tumors, no statistical difference was found compared to the administered dose. Moreover, no significant transformation of the particles, assessed via changes in the out-of-phase magnetic susceptibility temperature profile, may have occurred over time, indicating no aggregation and a negligible metabolization of the entrapped MNPs over three weeks (Figure S13b).

### *In Vivo* Testing of AMF-Tunability of Enzyme Prodrug Activation

**Figure 5:**
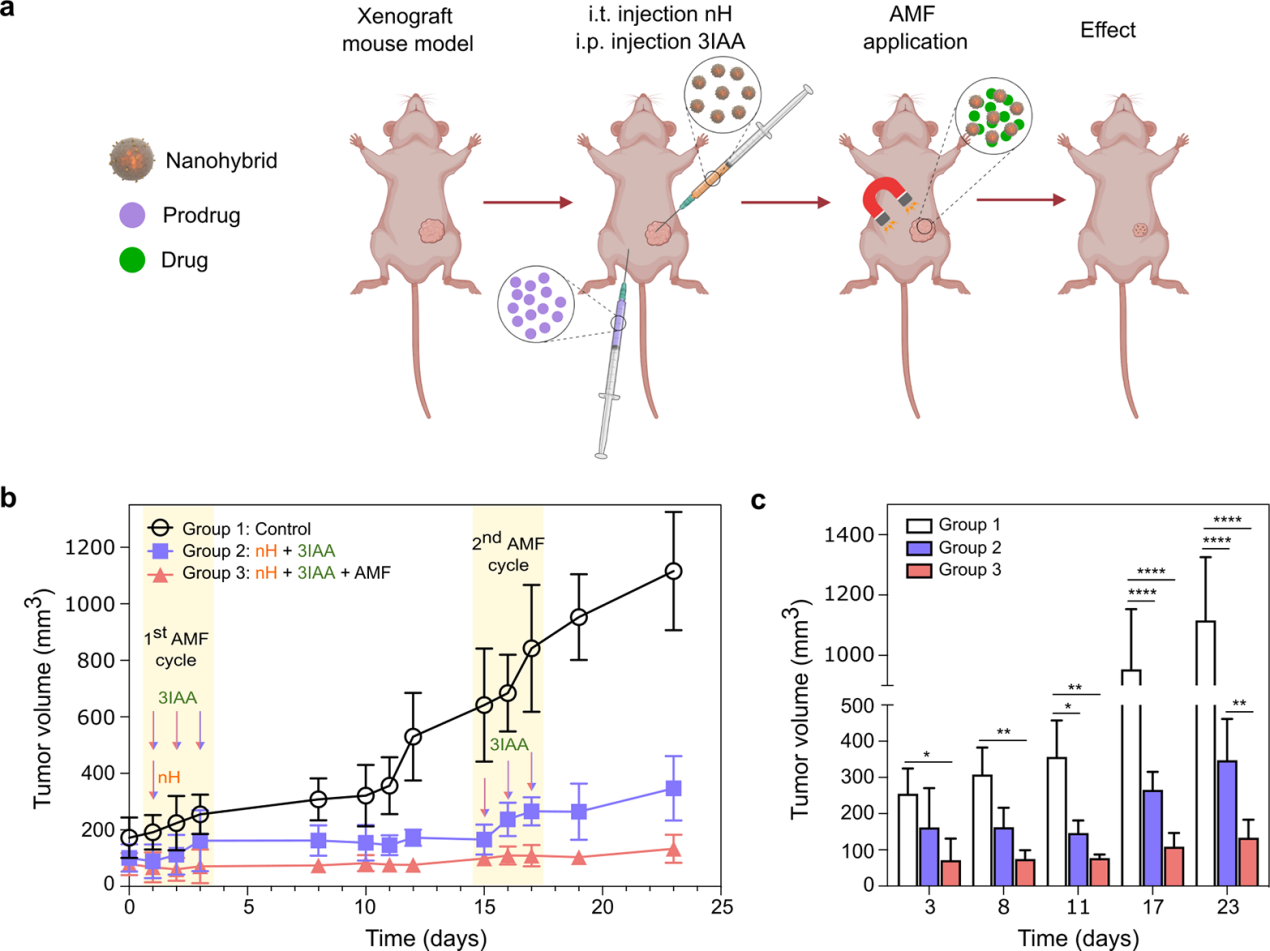
a, Schematic representation of enzyme therapy. b, Mice received a single intratumoral administration of nHs on day 1 followed by an intraperitoneal injection of the prodrug 3IAA. Just after the injections, mice were exposed to the AMF (*f* = 377 kHz; *H* = 15.9 kA/m; 5.9·× 10^9^ A/m·s) for 1 h. In the following two days, mice received only the intraperitoneal injection of the prodrug 3IAA and were exposed to AMF (Group 3) for 1 h at the previously mentioned conditions. After 15 days, the same prodrug/AMF cycle regimen was repeated with no additional administration of nHs. The six i.p. injections of prodrug and the single i.t. injection of the nHs carried out have been represented as arrows on the treatment timeline. The tumor volume progression is shown with tumor growth suppression effect in the nH + 3IAA + AMF group (Group 3). Data are shown as mean ± standard deviation (*n* = 4 per group). c, Statistical comparison of tumor volume at different time intervals of the experiment. Two-way ANOVA ****P < 0.0001, **P < 0.01, * *P* < 0.05.

The slow transformation of the nHs over time in physiological conditions (*in vitro* and *in vivo*) (Figure S11 and Figure S13) supports the use of a single dose of nHs followed by two cycles of treatment separated by 15 days. Each cycle consisted of the intraperitoneal administration (i.p.) of 3IAA and the immediate exposure to AMF for three consecutive days. We divided the mice bearing MIA PaCa-2 tumors into three groups: a control group without treatment (Group 1) that was i.t. administered with phosphate buffer; a second group in which nHs (23U·mL^–1^ and 2.25 mg·mL^–1^ Fe) were i.t. administered followed by an i.p. injection of the prodrug 3IAA (Group 2); and a third group identical to Group 2 but with AMF application (*f* = 377 kHz; *H* = 15.9 kA/m; 5.9 × 10^9^ A/m·s) after each 3IAA injection (Figure 5a and Figure S14a). A prodrug concentration of 200 mg·kg^–1^ was chosen according to previous studies described in the literature.^61,74^ As our *in vitro* results demonstrated that AMF did not induce cell death in nHs without prodrug (Figure 4f and Figure S12b), the *in vivo* experiments did not include this control. We have included the minimum groups of animals needed to evaluate the effect of AMF on prodrug conversion efficacy while complying with the 3R’s guidelines for Animal Research.

Weight loss was not observed in any mice from the three different groups (Figure S14b) which is an indicator usually used to discard general toxicity of the applied treatment. Moreover, no change in the global temperature of the tumors was observed *ex vivo* upon AMF application (Figure S14c). Therefore, from Figure 5b,c it is possible to conclude that specific tumor toxicity is triggered by *in situ* prodrug bioconversion of nHs, and it is clearly effective in slowing down tumor growth with respect to the nontreated control group.

Although in the nHs+3IAA-treated group without exposure to AMF cycles (Group 2) tumor growth was halted for 15 days, the second injection of 3IAA could not prevent tumor growth. However, tumor growth in the AMF-treated group (Group 3) was completely halted until the treatment was ended after 23 days from nH inoculation (Figure 5b,c). A mean increase in tumor volume up to 1000 mm^3^ among control mice was followed as the humane end point of the experiment.

The *in vivo* results are in line with the results observed *in vitro* and, thus, clearly support the feasibility of acquiring remote control over DEPT by means of magnetic heating. It is also important to point out that nH injection was performed once at the beginning of the treatment (day 1 in Figure 5b) which confirms as previously discussed that silica coentrapment ensured protection against degradation for both HRP and the coentrapped nanoactuators. This represents an added advantage of the proposed method for DEPT controllable by nanoactuation, as it could allow thinking of cyclic treatment schemes without the need for the short-term reinjection of the therapeutic nHs.

## Conclusions

The approach developed herein for the preparation of a nH biocatalyst provided excellent physicochemical and biological properties to be used in the development of alternative DEPT treatments with remote spatiotemporal control. Each of the components of the nanobiocatalysts played a crucial role in the final application of the nHs. Apart from the obvious conversion of 3IAA into cytotoxic species, we demonstrated that the HRP integrated into the nH was stabilized against denaturing agents and remotely activated upon exposure to AMF which converts the coentrapped MNPs into local hotspots. Cytotoxicity due to the prodrug bioconversion into toxic oxidative species was proven to be the sole consequence of the remotely triggered enzyme activity that caused cell death *in vitro*. Indeed, the mere exposure of cells to nHs and prodrug without the application of AMF or to nHs applying AMF did not result in a significant decrease in cell viability. Furthermore, we have proven the AMF-tunability and effectiveness of these prodrug-converting nHs *in vivo*. Tumor growth arrest was observed when the intratumorally injected nHs converted the intraperitoneally administered prodrug under AMF exposure. Indeed, our report herein describes the use of a remotely activated nH as an enzyme carrier system for DEPT. Moreover, our work shows the nanoactuation of enzymes not directly attached to MNPs through magnetic heating for a therapeutic application. As 3IAA is a poor substrate for mammalian peroxidases, triggering its remote site-specific oxidation by magnetic heating, as demonstrated here, holds the promise of producing the therapy *in situ* at the site of disease with little or no systemic exposure or side effects. Our findings could be extended to the use of thermophilic therapeutic enzymes as a strategy that allows one of the great limitations of current tumor prodrug enzyme therapies to be overcome, which is their off-target activation. This achieved milestone would surely help advance toward the implementation of biorthogonal catalysis to provide *in situ* production of the cytotoxic biologically active products by using specific exogenous enzymes as catalysts, thus focusing the treatment on the targeted site. Our studies encourage additional experimentation for a better insight into its biomedical potential and for bringing this nanodevice closer to its application in cancer treatment.

## Experimental Section

### Materials

Horseradish peroxidase Type VI (EC 1.11.1.7), polyethyleneimine (PEI) (MW 1300), 2,2′-azino-bis(3-ethylbenzothiazoline-6-sulfonic acid) diammonium salt (ABTS), indole-3-acetic acid (3IAA), hydrogen peroxide, d-(+)-maltose monohydrate type II, sodium phosphate dibasic, potassium phosphate, sodium borohydride 98%, 4–5-dihydroxy-1,3-benzenedisulfonic acid disodium salt monohydrate, tetramethyl orthosilicate (TMOS), and 16% formaldehyde (w/v), methanol-free (PFA 16%) Pierce were purchased from MERCK. d-Trehalose, ((3-(4,5-dimethylthiazol-2-yl)-2,5-diphenyltetrazolium bromide) MTT (Invitrogen), CM-H2DCFDA (Invitrogen), LIVE/DEAD Viability/Cytotoxicity Kit, for mammalian cells (Invitrogen), Alexa Fluor 488 Phalloidin (Invitrogen), and DAPI (4′,6-diamidino-2-phenylindole, dihydrochloride) (Invitrogen) were purchased from Fisher Scientific. Sodium acetic acid, sodium metaperiodate, sodium chloride, potassium chloride, potassium hydroxide (pellets) 85%, and glycerol PA-ACS-ISO were purchased from Panreac. Iron standard solution for AAS, 1 mg/mL Fe in 2% HNO_3_ was purchased from Across Organics. Gel filtration PD10-Columns were from GE Healthcare. Magnetic nanoparticles (MNPs) fluidMag-PAS were purchased from Chemicell MIA PaCa-2 (CRL-1420). Pancreas cancer cell line was purchased from ATCC. Advanced Dulbecco’s Modified Eagle’s Medium (DMEM, Gibco), phosphate buffer saline (PBS, pH 7.4, Gibco), Dulbecco’s phosphate-buffered saline (DPBS) (Gibco), Glutamax -I CTS (100X) (Gibco), and penicillin–streptomycin (100X) (Gibco) were purchased from Fisher Scientific. Trypsin was obtained from Sigma-Aldrich and Fetal Bovine Serum (Bio Whittaker) was purchased from Lonza.

### Oxidation of HRP

The oxidation was performed using a modification of Zalipsky’s PEGylation protocol.^75^ Briefly, HRP (3 mg) was dissolved in 1.8 mL of 10 mM sodium phosphate containing 154 mM sodium chloride, pH 7.2. Simultaneously, 8.6 mg of sodium periodate was dissolved in 200 μL of distilled water and protected from light. The sodium periodate solution was immediately added to the enzyme solution, and the sample was gently agitated. The 2 mL mixture was incubated in the dark for 1 h at 25 °C with constant end-over-end agitation. The reaction was quenched by the addition of 2.5 μL of glycerol (99.5%), and the oxidized enzyme was then purified by using a desalting PD10 column (GE Healthcare) equilibrated with 100 mM sodium phosphate pH 7.2 containing 154 mM sodium chloride. Oxidized HRP was concentrated to 1 mg mL^–1^ using Amicon Ultra-4 10K.

### Synthesis of Nanohybrids (nHs)

40 μL of HRPox (1 mg mL^–1^) and 50 μL of 10% polyethyleneimine (PEI) MW 1300 dissolved in water and adjusted to pH 8.0 were mixed in 0.4 mL of 5 mM sodium phosphate buffer containing 300 mM trehalose pH 8.0 and gently agitated in an end-over-end roller for 15 min at 25 °C. Then, MNPs (fluidMag-PAS) at a final concentration of 1 mg/mL were added, followed by the drop-by-drop addition of 100 μL of previously hydrolyzed TMOS solution prepared by diluting TMOS in hydrochloric acid (1 mM) to a final concentration of 1 M. This mixture was incubated for 30 min at 25 °C. The resultant entrapped HRP preparation was then centrifuged (12,225*g*) for 5 min and incubated with 0.1 M sodium phosphate containing 300 mM of sodium chloride pH 8.0. to remove non-entrapped and/or ionically adsorbed enzymatic molecules to the surface of the nHs for 15 min at 4 °C. Then, the mixture was washed three times with 0.1 M sodium phosphate buffer, pH 8.0. Post-entrapment reductive amination was performed to simultaneously obtain (i) a covalent three-dimensional rigidification of the entrapped HRP and (ii) functionalization of the nHs surface with functional glucose moieties. After the condensation of the silica and the subsequent washing steps, the nHs were then incubated in 0.4 mL of 25 mM sodium bicarbonate, pH 10.0, overnight at 4 °C to facilitate the formation of Schiff bases between the aldehyde groups generated in the sugars of the oxidized enzyme and unreacted primary amino group from PEI integrated into the silica matrix. The mixture was then centrifuged (12,225*g*) for 5 min and incubated in 0.4 mL of a solution of 1 M maltose dissolve in 25 mM sodium bicarbonate pH 10.0 containing 1 mg mL^–1^ of sodium borohydride on a roller shaker at RT for 1 h. As sodium borohydride reduces unstable Schiff bases into irreversible secondary amine bonds, it triggers both (i) the irreversible covalent 3D rigidification of the oxidized enzyme and (ii) the irreversible covalent binding of maltose by reductive amination between the aldehyde formed by the spontaneous ring opening of the maltose terminal reducing glucose unit with exposed primary amino groups of the nHs. The resultant nanoparticles, coentrapping HRP and MNPs, are noted as nHs. Upon several washes by centrifugation using 0.1 M sodium phosphate pH 8.0 buffer the nHs were stored at 4 °C until use.

### Standard Enzyme Activity Assay

The activity of the free and entrapped enzyme preparations was measured by a colorimetric assay using 9.1 mM ABTS, (εM = 36.8 mM^–1^ cm^–1^), as a substrate. The final assay contained 1.7 mL of 0.1 M potassium phosphate, pH 5.0, at 25 °C, 0.1 mL of 9.1 mM ABTS, 0.2 mL 0.3% (w/w) hydrogen peroxide solution (H_2_O_2_) in deionized water and 10 μL of the soluble or nH preparations. The oxidation of ABTS was measured in a spectrophotometer at a wavelength of 405 nm for 2 min (Agilent Cary 60 UV–vis). One enzyme unit (U) was defined as the amount of HRP able to oxidize 1 μmol of ABTS per mL in the conditions described above.

### Enzyme Entrapment Parameters

Immobilization yield (*I*) was expressed as the percentage of the ratio of the difference between the initial activity offered in the reaction and the activity recovered in the supernatant and the initial activity (eq 1).
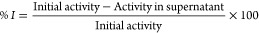
1

Expressed activity (EA) was defined as the percentage of the ratio between the activity immobilized onto the carrier and the activity offered to the carrier (eq 2).
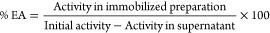
2

### Physicochemical Characterization of the nHs

Transmission and scanning electron microscopy (TEM and SEM, respectively), dynamic light scattering, zeta potential, infrared spectra (IR), and thermogravimetric analysis (TGA) validate that the nH was synthesized correctly. The morphology of the resulting nHs was characterized by scanning electron microscopy (SEM) in a field-emission FEI Inspect F operated at 10 kV using the Everhart–Thornley Detector (ETD) for back-scattered electron mode. Before being placed in the specimen holder, samples were washed three times in Milli-Q water to remove any salt or maltose excess and diluted to a final concentration of dry support of 0.2 mg/mL. The nHs suspension sample was allowed to dry at room temperature and deposited into a piece of conductive double-sided carbon tape, and it was sputter-coated with platinum before the examination. The particle-size distribution was evaluated from several micrographs of random regions using an ImageJ image analyzer, selecting approximately 100 particles for further consideration, which resulted in stable size-distribution statistics. The integration of the MNPs in the silica matrix and immunogold labeling were studied by transmission electron microscopy (TEM) in a Tecnai G2 TEM (FEI) operated at 200 kV and high angle annular dark field scanning transmission electron microscopy (HAADF-STEM) operated at 300 kV, respectively (see Supplementary Method 1). A coated copper grid was placed onto a drop of 0.6 mg/mL nH suspension and then dried overnight. Dynamic light scattering (DLS) and zeta potential measurements were performed in Milli-Q water and 1 mM potassium chloride, respectively, at a sample concentration of 0.04 mg mL^–1^ of dry support, respectively, on a Malvern Zetasizer, using ten runs per measurement and six measurements at 25 °C and pH 7. The surface chemistry and conjugation of different functional moieties were elucidated from FTIR analysis carried out using a (Jasco FTIR 4100) spectrometer recorded in the 400–4000 cm^–1^ wavenumber range, at a resolution of 4 cm^–1^. TGA was performed with TA STD 2960 simultaneous DTA-DTGA instrument in air at heating rates of 10 °C/min. Nitrogen adsorption isotherms were measured with N2 at 77 K on a Micromeritics ASAP 2020. Before the measurement, samples were lyophilized and then outgassed under vacuum at 383 K for 12 h. BET analysis was performed using the appropriate pressure range based on published consistency criteria. The micropore surface area was assessed with the t-plot method applied in the thickness range 3.5–6 Å.

### Magnetic Characterization of the Obtained nHs

A volume of the suspension of each sample, at a known iron concentration, was placed on a piece of cotton wool and allowed to dry at room temperature. The cotton piece was then placed in a gelatin capsule for magnetic characterization. The magnetic measurements were performed in a Quantum Design (USA) MPMS-XL SQUID magnetometer equipped with an AC (alternating current) magnetic susceptibility option. Both DC (direct current) and AC measurements were performed. For the DC measurements, field-dependent magnetization was recorded at 300 K with a maximum field applied of 2 T. These measurements allowed the evaluation of the magnetic properties of the material, in particular, to verify if the particles were superparamagnetic at room temperature, if their hysteresis loop was close, with negligible coercivity. These measurements also allowed comparing the saturation magnetization with that of the bulk material (76 Am^2^/kg Fe_2_O_3_ for maghemite),^51^ to evaluate if there was any effect of the particle size on the magnetic properties of the material. AC measurements were performed with an AC amplitude of 0.41 Oe, in the temperature range between 2 and 300 K, and at a frequency of 11 Hz. These measurements were useful to verify the aggregation of the particles and the impact that this process could have on their magnetic behavior.

### Quantification of Entrapped Fe in the nHs

Iron concentration was determined using a standard colorimetric procedure. An aliquot was digested with aqua regia for 15 min at 60 °C and diluted with Milli-Q water. A calibration curve was prepared by dilution of an iron standard solution of 1 mg mL^–1^ of Fe in 2% HNO_3_. The digested samples were incubated at room temperature for 15 min after the addition of KOH (4 N), 4,5-dihydroxy-1,3-benzenedisulfonic acid disodium salt monohydrate (Tiron), and sodium phosphate buffer (0.2 M, pH 9.7). Finally, sample absorbance (480 nm) was measured on a UV/vis spectrophotometer (Thermo Scientific Multiskan GO MA, USA) and compared to the calibration curve.

### Temperature Profile

To study the optimum temperature, the activities of soluble and entrapped HRP were measured at various temperatures (20–60 °C) for 10 min under standard assay conditions, changing the temperature of incubation using a thermoblock as a global heating source.

### Thermal Stability

The thermal stability study was carried out at 50 °C, wherein aliquots of soluble and entrapped HRP suspensions in 0.1 M sodium phosphate buffer, pH 8.0, were withdrawn at different time intervals. Their residual activity was measured using the previously described standard activity assay using a final assay concentration of 0.7 U/mL. Residual activity was defined as eq 3:
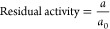
3where *a* is the activity at the selected time point and *a*_0_ is the initial activity. Biocatalyst inactivation was modeled based on the deactivation theory proposed by Henley and Sadana.^76^

Inactivation parameters were determined from the best-fit model of the experimental data, which was the one based on one-stage and two-stage series inactivation mechanism without residual activity for soluble and entrapped HRP, respectively, using the software GraphPad Prism 9 (San Diego, CA, USA). E0, E1, and Ed are the corresponding enzyme species of progressively less specific activity. E0 is the initial active enzyme, and it is deactivated to E1. Subsequently, E1 is inactivated to Ed, which is the nonactive form of the enzyme. The first-order transition rate constants of the two inactivation steps are *k*_1_ and *k*_2_. Experimental data were fitted in the proposed model and inactivation parameters (*k*_1_ and *k*_2_) for HRP and nHs were determined.

The stability factor (SF) was the parameter used for a quantitative comparison of the stability of the biocatalysts and is defined by eq 4:
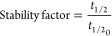
4where *t*_1/2_ is the half-life of the more stable sample and *t*_1/2_0__ is the half-life time of the less stable sample. Half-life time (the time at which the residual enzyme activity is half of its initial value) was determined by interpolation from the respective model.

### Magnetic Heating Properties of MNPs

The heating capabilities of bare and entrapped MNPs under the application of an external AMF were determined by the specific loss power (SLP) parameter (also referred to as specific absorption rate (SAR)), which provides a measure of the rate at which energy is absorbed per unit mass of the magnetic nanoparticles (eq 5).
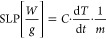
5where *C* is the specific heat capacity (*e.g*., water 4185 W·s/l·K) (assuming 1 L = 1 kg water), (d*T*/d*t*) is the heating rate in Kelvin per second, and *m* is the concentration of the nanoparticles in g/L.

A 1 mL sample at a concentration of 0.9 mg Fe/mL dispersed in water was used to determine the heating capacity of the MNPs. The global heating profiles of the MNPs samples were recorded using a fiber-optic probe provided with the AMF applicator (nB nanoscale Biomagnetics D5 series) used to apply an AMF with an amplitude of 36 mT and a frequency of 763 kHz. The CAL-2 coil with the capacitor 120 nF was used for these calorimetric measurements. Calculations were performed with the first 10 s within linearity.

### Activation of the Entrapped Enzyme and Determination of the Local Temperature Triggered by Magnetic Heating

AMF application was carried out at a frequency of 763 kHz with an amplitude of 36 mT using the CAL-2 coil with the capacitor 120 nF of the D5 series magnetic heating system (nB nanoscale biomagnetics, Zaragoza, Spain). At this condition, the activity of the nH using the standard activity assay already described at a final assay concentration of 0.15 U/mL was measured for 5 min while AMF was either applied or not. Similarly, the activity of the nH, using the same conditions, was measured at different temperatures ranging 20–70 °C using a thermoblock as a global heating source. The temperature dependence of the rate constant (below the inactivation temperature) was calculated using the Arrhenius equation (eq 6).
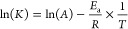
6where *K* is the rate constant, *T* is the absolute temperature, *A* is the pre-exponential factor, *E*_a_ is the activation energy for the reaction, and *R* is the universal gas constant.

### Thermal Stability of the Entrapped Enzyme upon AMF Application

An AMF with a frequency of 763 kHz and an amplitude of 36 mT was applied using the CAL2 coil of the D5 series magnetic heating system (nB nanoscale biomagnetics, Zaragoza, Spain) to a nHs dispersion (0.7 U/mL) prepared in 0.1 M sodium phosphate buffer, pH 8.0. Upon AMF application for different time intervals, the activity of the nH was determined using the standard activity assay described.

### Cell Culture Conditions Used for *In Vitro* Studies

MIA PaCa-2 cells (human pancreatic carcinoma, CRL_1420TM) were cultured at 37 °C in a 5% CO_2_ atm in complete Advanced Dulbecco’s Modified Eagle Medium (cDMEM; Gibco, Thermo Fisher Scientific) supplemented with 10% fetal bovine serum (FBS, BioWhittaker), 2 mM glutaMAX TM, and 100 U/mL of penicillin/streptomycin (Gibco, Thermo Fisher Scientific). Cells were confirmed to be free of mycoplasma and endotoxin contamination (see Method S2, Table S5).

### nHs Internalization Studies

Confocal microscopy sections were used to assess the interaction and internalization of the nHs. MIA PaCa-2 cells were grown in slide 8 well-plate (SPL Life science) at 60,000 cells/well for 24 h. Then, cells were incubated with 200 μg mL^–1^ for 4 or 24 h. After incubation, cells were washed with DPBS (PBS with additional Ca^2+^ and Mg^2+^) and fixed with 4% paraformaldehyde (PFA, 15 min). Cells were further stained with Alexa Fluor 488 Phalloidin and DAPI according to the manufacturer’s instructions. Finally, the samples were mounted in ProLong Gold (ThermoFisher) and images were acquired on a confocal Zeiss LSM 880 with a Plan-Apochromat 63X/1.4 oil (DT 0.19), DIC objective.

### Stability of nHs in Complex Media

The stability of the three main components of the nanosystem was assessed in complex media for 23 days at 37 °C in cDMEM or in Artificial Lysosomal Fluid (ALF). nHs with the entrapped HRP and its soluble counterpart (16 U/mL) were incubated in each assessed condition. Samples were withdrawn at different time points or end points and analyzed by measuring (i) relative enzyme activity (see Standard Enzyme Activity Assay), (ii) silica degradation by TEM (see Physicochemical Characterization of the nHs), and (iii) MNPs degradation by the temperature dependence of the AC magnetic susceptibility (see Magnetic Characterization of the Obtained nHs). Prior to being exposed to ALF, the nHs were incubated for 24 h in 10% serum to mimic the nH interactions with serum proteins occurring before cell internalization (data shown in Figure S11a).

### Inherent nHs Cell Cytotoxicity Determined by MTT Assay

The MTT (3-(4,5-dimethylthiazol-2-yl)-2,5-diphenyltetrazolium bromide) assay was performed to assess the viability of MIA PaCa-2. Briefly, 5000 cells were seeded in a 96-well plate (three replicates per sample) and incubated for 48 h before exposure to different concentrations of nH (1000; 500; 250; 125; and 62.5 μg mL^–1^). Cells were further incubated with 0.25 μg mL^–1^ of MTT dye (in DMEM) at 37 °C. After 1 h, the plate was centrifuged using an Eppendorf centrifuge 5810R with an A-4-62 rotor at 1250 rpm for 20 min. The formazan crystals were dissolved with 200 μL of dimethyl sulfoxide. The absorbance was measured in the Thermo Scientific Multiskan GO microplate reader at 540 nm. The relative cell viability (%) was determined by normalizing the absorbance readings from the treatment groups to the control group. Experiments were performed in triplicates, and data are represented as the mean value ± the standard error of the mean.

### *In Vitro* Assessment of the Biological Response Triggered by AMF-Mediated Prodrug Bioconversion Determined by Several Biological Assays

The biological response of MIA PaCa-2 cells upon triggering prodrug bioconversion by AMF application was analyzed using different biological assays. In all cases for the AMF-triggered treatment, 5000 cells were seeded in a 96-well plate (three replicates per sample) and incubated for 48 h in cell culture media. Then, cells were exposed to 125 or 62.5 μg ml^–1^ of nH and 2 mM of 3IAA. Immediately after nH and prodrug addition, cells were placed at the planar coil (PC^90^) of the D5 series magnetic heating system (nB Nanoscale Biomagnetics) where they were exposed or not for 1 h to AMF (424 kHz and 20 mT) at RT using. Several controls have been performed under the same experimental conditions and their biological response determined irrespective of whether the AMF was applied or not: (i) cell samples to which no nHs were added (control of a possible direct effect of applying AMF to cells); (ii) cell samples to which nHs was added but not the prodrug (control of a possible direct effect of the magnetic heating triggered by the entrapped MNPs within nHs when AMF was applied). After the AMF-mediated treatment was performed, cell samples were put back in the cell incubator at 37 °C and 5% CO_2_, and different biological assays were further carried out to assess the biological response elicited.

#### a MTT Assay

After the AMF-mediated treatment, cells were further incubated for 24 h before the MTT assay was performed as already explained above. Experiments were performed in triplicate, and data were represented as the mean value ± the standard error of the mean (S.E.M.).

#### b Live/Dead Assay

After AMF-mediated treatment, cells were further incubated for 24 h. Then, the media was removed and 100 μL of a working solution of DPBS (PBS with additional Ca^2+^ and Mg^2+^) containing 2 μM of calcein AM and 4 μM EthD-1 were added to stain viable and nonviable cells, respectively. After 45 min of incubation, cells were imaged under the inverted fluorescence microscope (Nikon Eclipse TE2000-S microscope) equipped with a digital camera (slight ds-Fi1c). Experiments were performed in duplicate. Live/Dead assay gives simultaneous indications of the biochemical and physical properties of cells by combining the use of two-color fluorescent dyes. Calcein AM stains live cells (in green) as it is converted from a permeable nonfluorescent into a cell-impermeant fluorescent analog by intracellular esterases. In contrast, ethidium homodimer (EthD-1) stains dead cells (in red), by producing red fluorescence by only binding to nucleic acids of cells with damaged membranes.

#### c Reactive Oxygen Species (ROS) Assay

Both intracellular and extracellular ROS were detected using a commercial ROS assay kit using the CM-H2DCFDA probe. After 6 h following the AMF-mediated cell cytotoxicity treatment previously described, 100 μL of a working solution of PBS containing 2 μM of dye was added to cells and incubated for 30 min. Cells were finally analyzed in a CytoFlex Flow Cytometer (Beckman Coulter). Experiments were performed in duplicate, and data were represented as the mean value ± the standard error of the mean (S.E.M.).

### Generation of MIA PaCa-2 Xenograft Mouse Model

Male athymic nude mice (Hsd: Athymic Nude-Foxn1^nu^), 4 weeks old, received a subcutaneous injection into the right flank with the human pancreatic cancer cell line MIA PaCa-2 suspended in 0.05 mL of sterile DMEM culture medium without phenol red using a 25 G needle. During the cell injection, animals were anesthetized by inhalation of isoflurane (4% for the induction step and 2% for maintenance). The mice were commercially obtained from Envigo and were maintained in the animal facilities of the CIBA (IACS-Universidad de Zaragoza). Before any procedure, mice were held one week after arriving from the animal facilities for acclimation. Animals were maintained according to the institutional animal use and care regulation of the Centro de Investigaciones Biomédicas de Aragón (CIBA, Zaragoza, Spain). All animal experiments were conducted according to the law RD53/2013 and approved by the Ethics Committee for animal experiments from the University of Zaragoza which is an accredited animal welfare body.

### Biodistribution and Metabolism Analysis of the nHs Using AC Magnetic Susceptibility Measurements

AC magnetic susceptibility measurements were selected for this purpose as this technique has been validated to distinguish between the endogenous iron (*e.g*., ferritin) and the iron originating from the MNPs allowing their detection with high sensitivity and specificity. This technique has also been validated to track small reductions in the average particle size or aggregation states.^77^ Mouse tissues (spleen, liver, and tumor) were freeze-dried overnight, and all the organs (except the liver) were transferred directly to gelatin capsule sample holders for magnetic characterization. Given the large volume of the liver, this organ was ground in a mortar to obtain a homogeneous powder. An aliquot (∼100 mg) of this powder was then placed inside a gelatin capsule for magnetic characterization. The temperature dependence of the AC magnetic susceptibility was measured using a QuantumDesign MPMS-XL SQUID magnetometer, using the AC option, a field amplitude of 4.1 Oe, and a frequency of 11 Hz.

### Magnetic Hyperthermia Treatment for Prodrug Conversion *in vivo*

Three weeks after cell inoculation, when the tumor size was about 100–150 mm^3^, mice were divided randomly into three different groups (Group 1: Control; Group 2: nH+3IAA; Group 3: nH+3IAA+AMF). Mice were intratumorally (i.t.) administered 1.15 U nH using a 30 G needle. The same day of the nHs injection and the following two days, mice were intraperitoneally (i.p.) injected with the prodrug 3IAA at 200 mg·kg^–1^ concentration. Just after the i.p. injections, mice were exposed to the AMF (*f* = 377 kHz; *H* = 15.9 kA/m; 5.9 × 10^9^ A/m·s) for 1 h. For these studies, an AMF-applicator specifically designed for *in vivo* experimentation was used (D3 Series, Nanoscale Biomagnetic SL) which operates with an integrated open coil for AMF generation similar to the coil of the AMF-applicator used for *in vitro* cell assays (D5 Series, NanoscaleBiomagnetics).

After 15 days, mice were again i.p. injected with the 3IAA and exposed to the AMF/prodrug cycle previously used. Mice were anesthetized with isoflurane and maintained during the AMF exposure onto a hot water bath system that prevents the mice from suffering hypothermia. One mouse belonging to the AMF-treated group was excluded due to conditions unrelated to the therapy. After the last AMF exposure, mice were maintained to evaluate the response to the treatment. Body weight and animal behavior were monitored every other day and tumor dimensions (length and width) were measured with a digital caliper. Tumoral volume was calculated as (*L* × *W*^2^)/2). Following the completion of treatment, animals were sacrificed, and the tumors were excised.

### Statistical Analysis

All data were expressed as mean ± SD of a minimum of three biological replicas. The statistical significance of the difference in means was evaluated using GraphPad Prism v 9.00. Two-way ANOVA and one-way ANOVA tests were used for the analysis of the data. The confidence interval was 95%.

## Supporting Information Available

Supplementary methods, figures, and tables, mainly including reductive amination of nHs, key factors for nHs synthesis, graphical scheme depicting the immunogold assay, physicochemical characterization of the obtained nHs, magnetic heating theory, inferring *T*_LOC_ in nHs upon AMF application from Arrhenius linearization plot, AMF stability of nHs, effect of the obtained nHs at the cellular level, nH stability in complex media, effect of the obtained nHs *in vivo*, and HRP entrapment parameters (PDF)
