# Supplementary material for: Remote Activation of Enzyme Nanohybrids for Cancer Prodrug Therapy Controlled by Magnetic Heating
Source: ACS Nano. 2023 Jun 26;17(13):12358–73. doi: 10.1021/acsnano.3c01599 (PMC10339790; doi:10.1021/acsnano.3c01599)
Supplement: Supplementary file 1 — nn3c01599_si_001.pdf [file nn3c01599_si_001.pdf]

Supporting information

Remote activation of enzyme nanohybrids for cancer prodrug therapy controlled by magnetic heating

Beatriz Torres-Herrero[1], Ilaria Armenia[1], Maria Alleva[1], Laura Asín[2], Sonali Correa[3], Cecilia Ortiz[3], Yilian Fernández-Afonso[1,2,4], Lucía Gutiérrez[1,2,4], Jesús M. de la Fuente[1,2], Lorena Betancor*[3], Valeria Grazú*[1,2].

[1]Instituto de Nanociencia y Materiales de Aragón (INMA), CSIC-Universidad de Zaragoza, Zaragoza 50009, Spain.

[2]Centro de Investigación Biomédica en Red de Bioingeniería, Biomateriales y Nanomedicina (CIBER-BBN), Madrid 28029, Spain.

[3]Laboratorio de Biotecnología, Universidad ORT Uruguay, Montevideo 11100, Uruguay.

[4]Departamento de Química Analítica, Universidad de Zaragoza, Zaragoza 50009, Spain

* betancor@ort.edu.uy;

*vgrazu@unizar.es

Method S1. Preparation of nanohybrids (nHs) sections by Tokuyasu technique and immunogold labeling. The nHs samples (1.3 mg mL^{-1}) were washed three times in PBS for 5 min and rinsed with glycine (0.15 % in PBS) for 10 min. 1.5 mL of gelatin (1 % in PBS) was added to change surface tension, and the sample was pelleted by centrifugation and transferred to 1mL of 12 % gelatin in PBS at 37 ˚*C* for 15 min. The sample was further centrifuged and then solidified in ice for 30 min.

Convenient blocks (about 1 mm^3) were prepared with a razor blade and immersed overnight in 2.3 M sucrose in rotating vials at 4 °C. Then, they were mounted on specimen holders and sectioned in cryogenic conditions using the cryo-ultramicrotome Leica EM UC7-FC7. Ultrathin sections of 70 nm thick were cut at -110 ˚*C* (blocks were first trimmed at -80 ˚*C*). To prevent stretching, sections were picked up from the diamond knife using a 1:1 mixture of 2.3 M sucrose in 0.1 M PBS and 2 % methyl cellulose and transferred to carbon-coated copper grids. Before TEM observation, grids were washed in a drop of fresh PBS for 30 min at 37 ˚*C,* and five subsequent washing steps in distilled water for 1 min each were performed.

For their immunolabeling, grids were first washed three times in a drop of glycine (0.15 % in PBS) for 2 min, then blocked with BSA (1 % in PBS) for 5 min and incubated afterward with 5 µL of 6 nm Colloidal Gold AffiniPure Goat Anti-Horseradish Peroxidase (EM Grade) (123-195-021) (Jackson ImmunoResearch) diluted in BSA (1 % in PBS) for 60 min. Next, the grids were passed over a series of droplets of PBS (5 x 2 min), postfixed in GA (1 % in PBS) under the fume hood for 5 min. Grids were washed 3 times in PBS for 2 min and 6 times in distilled water for 1 min. Eventually, grids were dried with filter paper and kept in the grid box at 4 ˚*C* until HAADF-STEM observation.

Method S2. Determination of endotoxin concentration by Limulus Amoebocyte Lysate assay in nHs. The presence of endotoxin or lipopolysaccharides (LPS) components

of Gram-negative bacterial cell walls on inhaled nanoparticles could cause immuno-toxicity such as fevers and disease in case of *in vivo* application or false biological responses to these particles using *in vitro* systems. Consequently, contamination by endotoxins would confound the result of tests in vitro. Therefore, the preliminary detection of endotoxins is required to confirm the insignificant levels of endotoxins in the test sample or for the adequate interpretation of data obtained by *in vitro* biological test systems.

50 μL of Endotoxin Standard dilutions and 25 μL of samples (ENM suspensions at 200 μg/ml or 20 μg/ml) were added to a pre-equilibrated plate at 37 ˚*C*. 25 μL of Endotoxin-Free Water is added to normal samples (final concentration of the sample 100 μg/ml or 10 μg/ml) and 25 μL Endotoxin Standard 1 to spiked samples. Keeping the plate at 37 ˚*C*, 50 μL of the reconstituted Amebocyte Lysate Reagent were added per well. Once the Amebocyte Lysate Reagent had been added to the plate wells, the plate was briefly removed from the plate heater and mixed by gently tapping 10 times on its side, avoiding spilling. The plate was returned to the plate heater and incubated at 37 ˚*C* for the time T1 indicated on the lysate vial. The chromogenic substrate was reconstituted with 3.4 mL of endotoxin-free water and warmed to 37 ˚*C* for 5 minutes before use. After an exact time T1, 100 μL per well of warmed reconstituted chromogenic substrate solution was added. Once the substrate solution had been added to all plate wells, the plate was briefly removed from the plate heater and tapped 10 times to facilitate mixing. It was then returned to the plate heater at 37 ˚*C* for T2. Then, 50 μL per well of stop solution (25 % acetic acid) were added. The plate was then removed from the plate heater and mixed by gently tapping 10 times on it. The optical density (OD) was read at 405 nm immediately after assay completion.

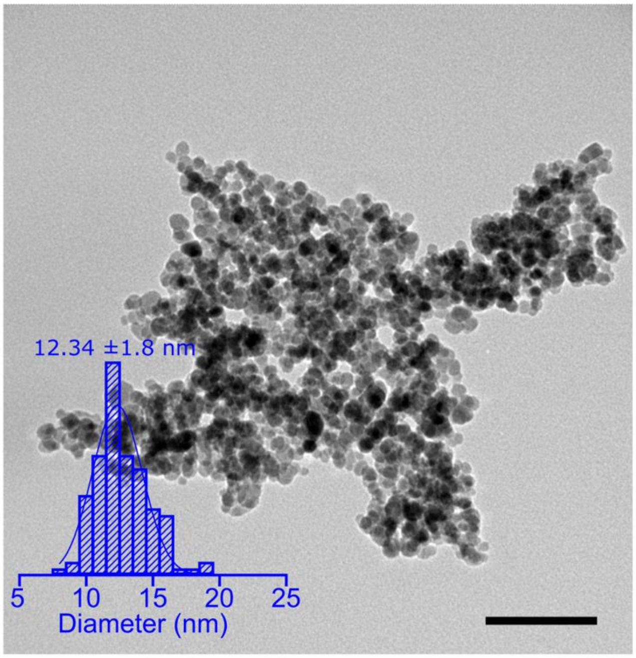

Figure S1. MNPs (FluidMAG-PAS) were used to prepare the developed nHs. a, Transmission electron microscopy (TEM) image and size distribution (n=120). Scale bar 100 nm.

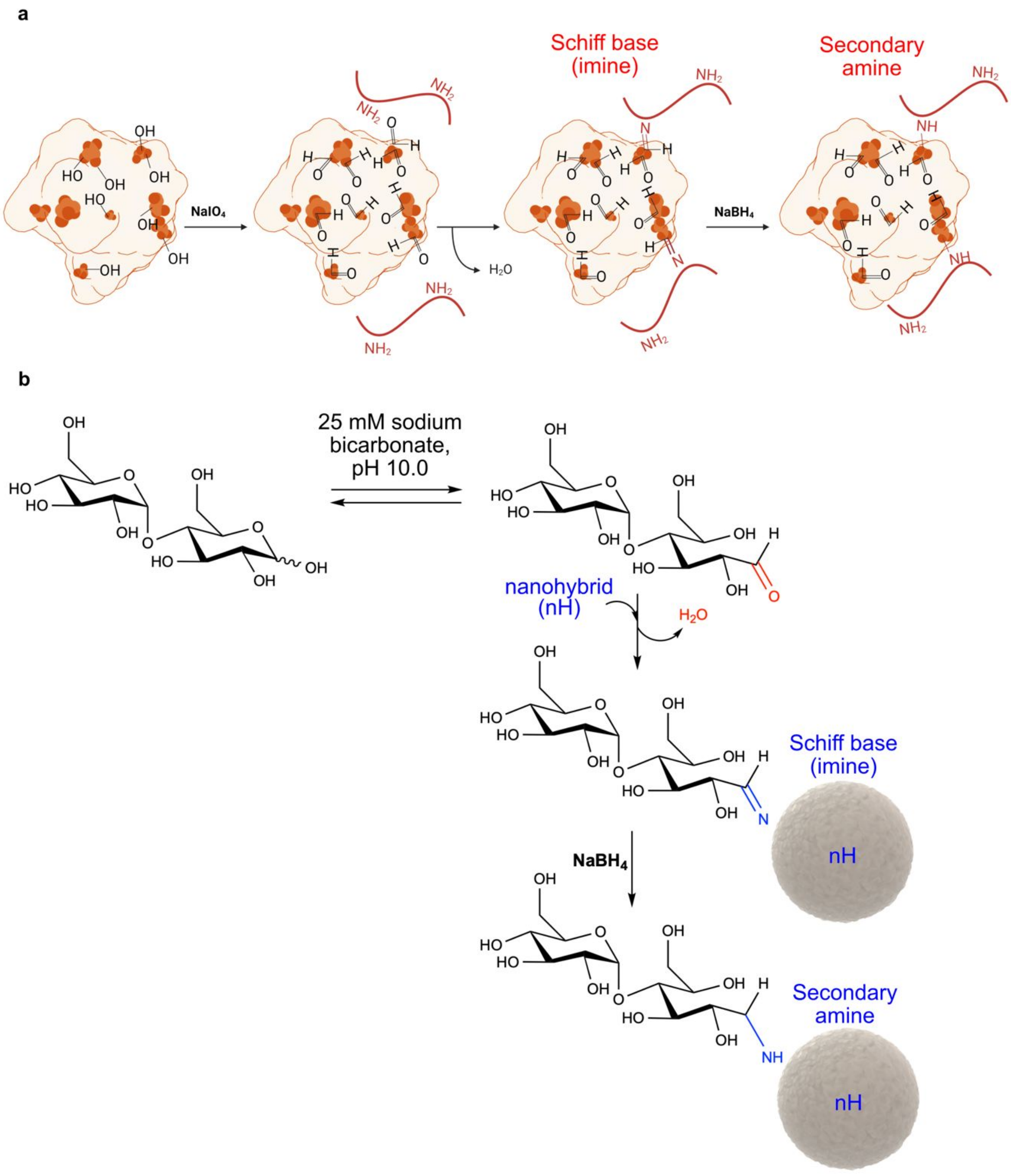

Figure S2. Reductive amination of nHs resulting in two different simultaneous covalent reactions: a, 3D-covalent matrix formation. Primary amines (R-NH_2) of the PEI, used to trigger silicification, undergo nucleophilic addition with aldehydes of the HRP´s oxidized sugar moieties generating unstable imine bonds (Schiff bases) that are, then, further reduced

to form stable covalent bonds. b, Maltose functionalization of nHs surface. Upon addition of maltose, the exposed primary exposed amino groups of nHs undergo nucleophilic addition with the aldehyde formed by the spontaneous ring opening of maltose´s terminal reducing glucose unit.[1] Once both imine bonds (a and b) were formed, a reduction step using sodium borohydride as a reductant allows the transformation of these reversible Schiff bases into irreversible secondary amine bonds. Image created with Biorender. Modeling of the nHs prepared by Blender and 3D Protein Imaging Studio.[2] 3D structure of HRP (PDB entry 1HCH) was rendered with 3D Protein imaging.[2]

a

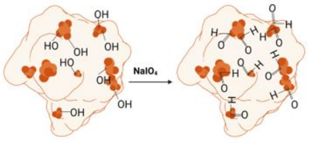

	nH (with HRP no oxidized)	nH (with HRP oxidized)
% Immobilization	30 ± 2	68 ± 3
% Expressed activity	4.5 ± 0.5	60 ± 7

b

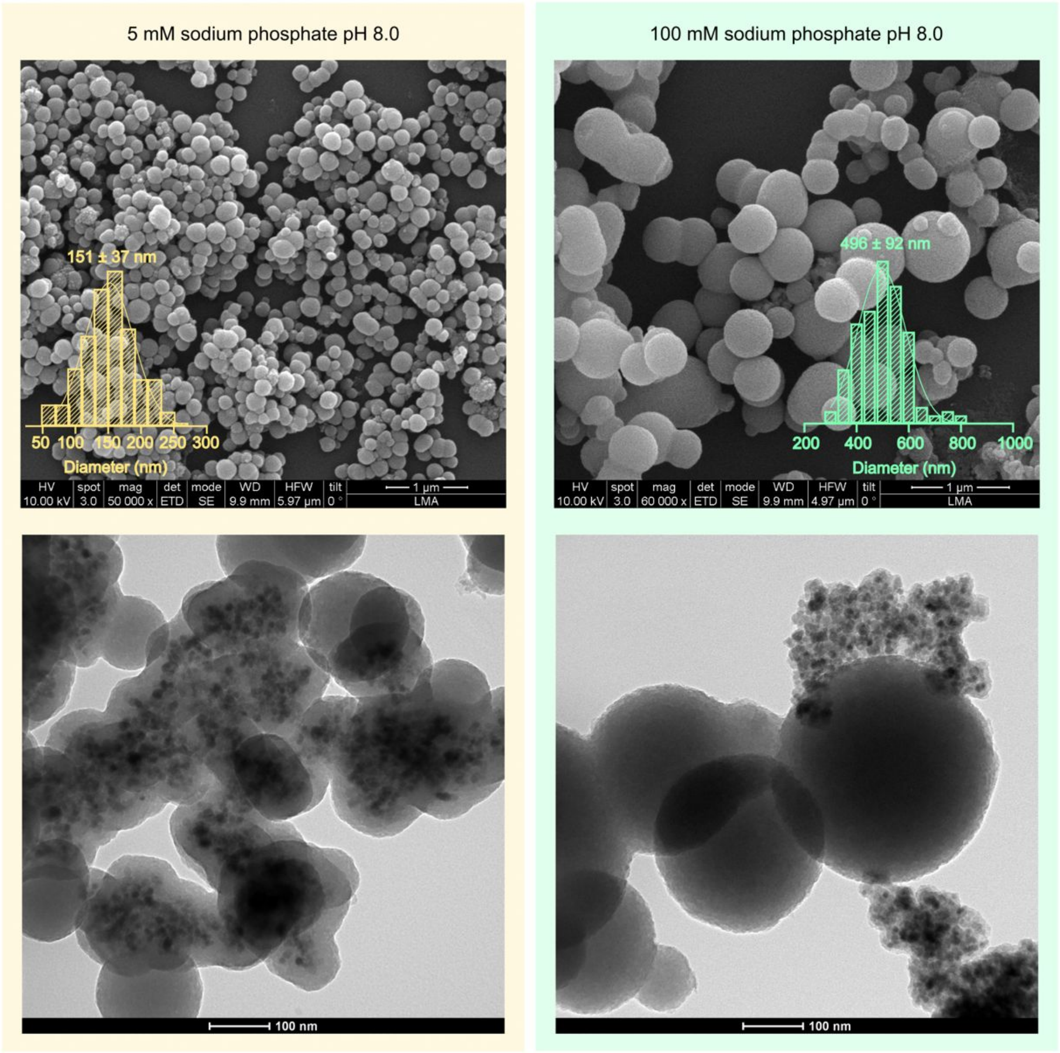

Figure S3. Key factors for nHs synthesis. a, Influence of oxidation of HRP sugar residues (in red) in the entrapment efficiency and expressed enzyme activity of the nHs. 3D structure of HRP (PDB entry 1HCH) was rendered with 3D Protein Imaging[2].

b, Influence of phosphate concentration in the final size of the obtained nHs: SEM and TEM images of the obtained nHs at 5 mM (left) and 100 mM (right) phosphate concentration.

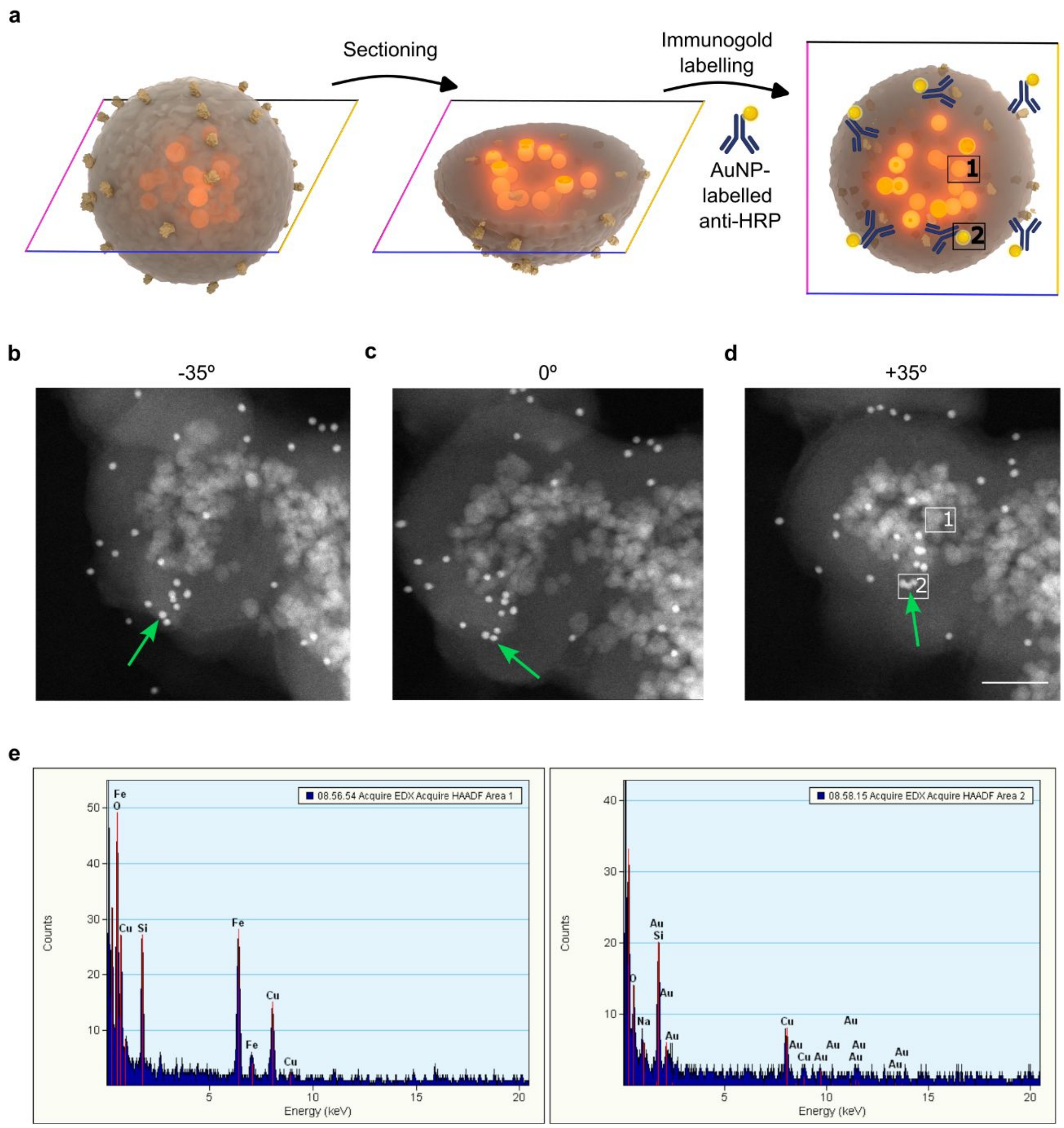

Figure S4. a, Graphical scheme depicting the immunogold assay for the colocalization of the HRP enzyme and the MNPs within the silica matrix. Modeling of the nHs prepared by Blender and 3D Protein Imaging Studio.[2] b,c,d, High Angle Annular Dark Field Scanning

Transmission Electron Microscopy (HAADF-STEM) images of nanohybrids' sections at different tilt angles of the sample holder. An ultrathin section of the nHs embedded in gelatin and immunolabeled using the methodology described as Method S1., was tilted in the TEM microscope. Projections were recorded at different viewing angles (a) -35°, (b) 0°, and (c) +35°. This procedure allowed studying the in-plane and out-of-plane position of HRP within the silica matrix. The green arrow showed the position at the tilting different angles of the same gold NP that immunolabeled the location of an HRP molecule. Scale bar: 50 µm. e, EDX spectra based on the HAADF-STEM mapping of the nHs section. The analyzed areas were represented by squares in (d) and in Figure 1d. i) *Area 1*: the area where MNPs are located; ii) *Area 2:* the area where AuNPs immunolocalized HRP molecules are located.

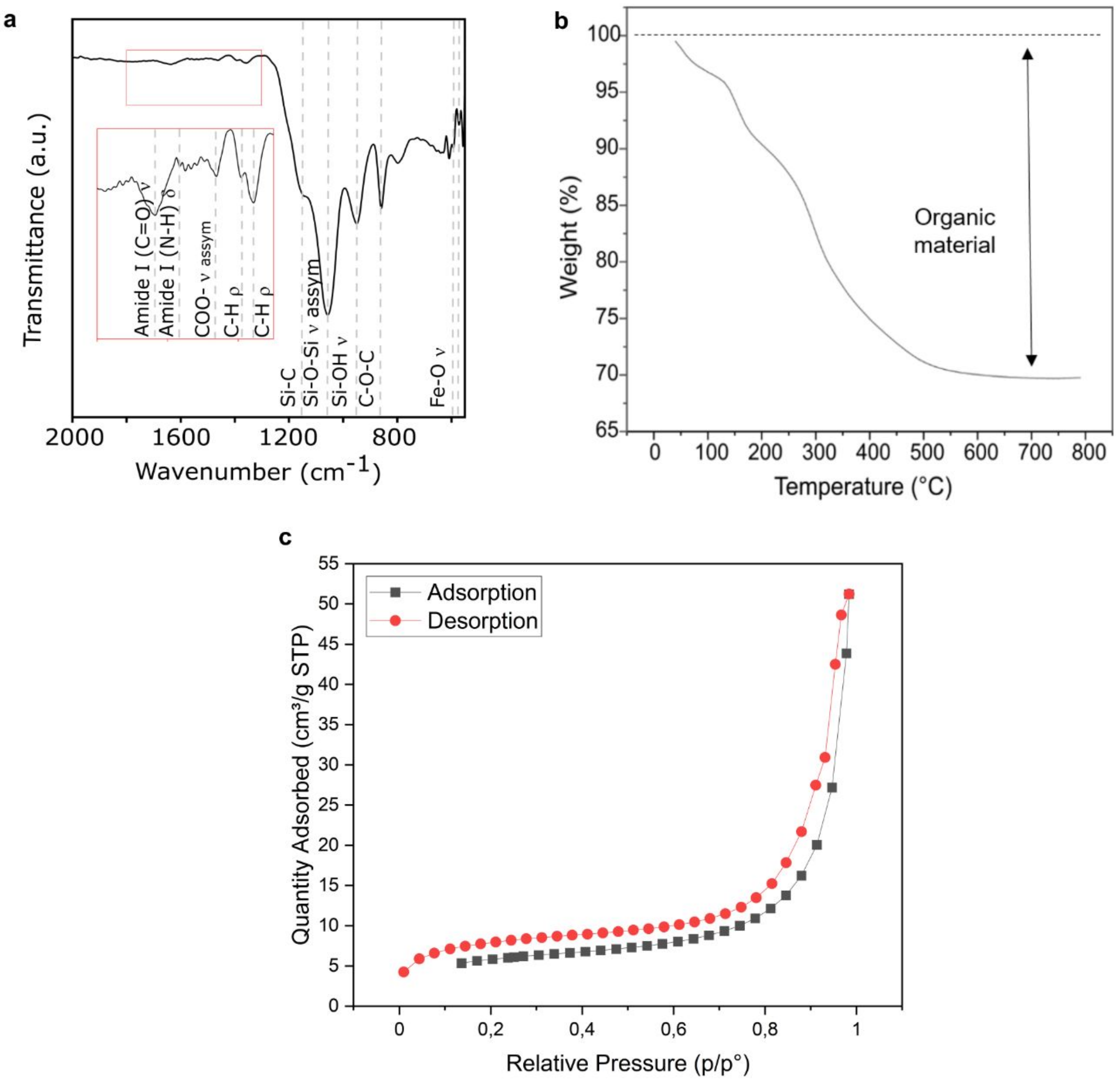

Figure S5. Physicochemical characterization of the obtained nHs. a, FTIR spectra. The red insert zooms the spectra zone that confirms the covalent interaction between the enzyme and the free amino groups of the Si matrix integrated PEI. b, TGA thermogram of nHs. c, Nitrogen adsorption/desorption isotherm determined by gas adsorption using N_2 at 77 K.

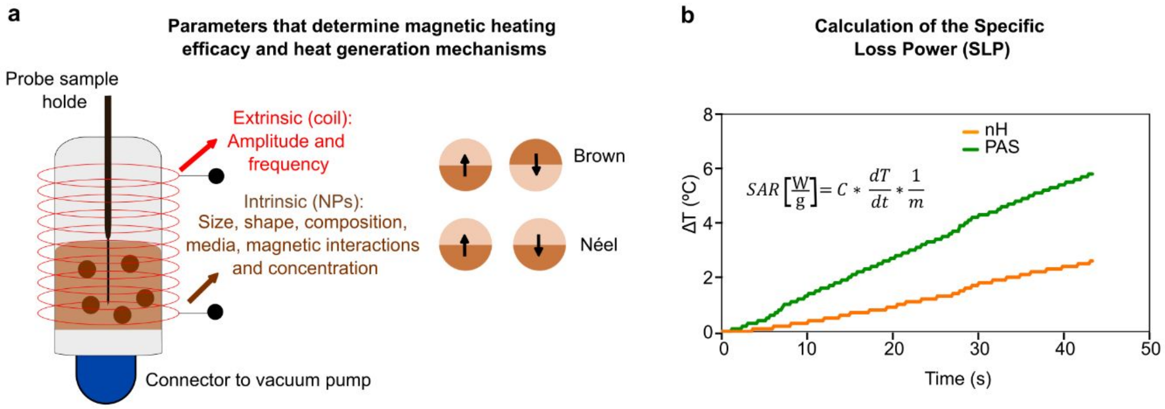

Figure S6. Magnetic heating capacity of MNPs. a, Scheme representing magnetic heating mechanisms and how to measure SLP. The mechanisms associated to release heat by MNPs exposed to an AMF are mainly described by Néel or Brown relaxation. In both cases, the magnetic moment of the particle reorients following the direction of the magnetic field. In the Brown mechanism, the magnetic moment is fixed within the particle, so the whole particle must rotate to be oriented in the field direction. In the Néel relaxation mechanism, there is no physical rotation of the particle, but the magnetic moment fluctuates within the crystal lattice of the MNPs. This AMF-triggered magnetic heating capacity of MNPs, either isolated or entrapped within the nHs, was calculated by determining their Specific Loss Power (SLP) based on the analysis of the initial slope of the heating curve in calorimetric experiments. For this, each NP suspension was placed within the coil that generates the AMF, and the temperature increment in the media where the NPs were suspended was recorded using an optical temperature probe. b, Heating curves of isolated (green curve) or entrapped (orange curve) MNPs diluted in water at the same concentration of Fe (0.9 mg mL^{-1}) were measured while an AMF of 763 kHz and 36 mT was applied. The slope obtained in the initial time frame, where heat losses are considered negligible, was used to obtain the SLP values reported in Figure 2a. This AMF setting corresponds to the maximum applicable in the AMF

applicator used, having been selected to maximize, and thus to be able to detect differences in their SLP values.

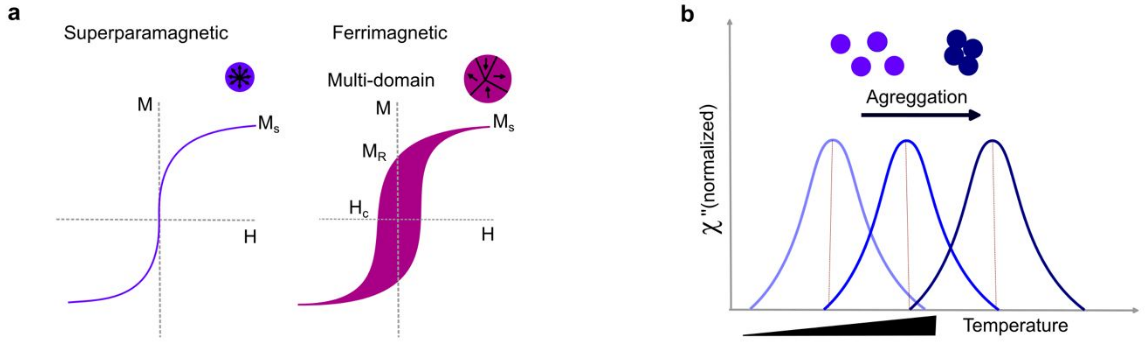

Figure S7. Magnetic characterization of MNPs. a, Schematic representations of magnetic hysteresis loops of superparamagnetic (violet) and ferromagnetic (purple) MNPs. Ferrimagnetic particles are divided into magnetic domains in which the magnetization has different directions (multidomain NPs). However, when the size of particles is reduced to the nanoscale, particles with a single domain appear. For even smaller sizes, the sample net magnetic moment becomes zero when the applied magnetic field is zero (superparamagnetic NPs). Thus, a way to differentiate between ferrimagnetic and superparamagnetic NPs is to plot their magnetization (M) variation with the magnetic field (H). In this hysteresis loop, the saturation magnetization (M_S) is reached when the H is strong enough to align the magnetic moments of the material. In multi-domain particles, once the M_S is reached, if the field is reduced to zero, the magnetization will not go to zero; in fact, a remanence (M_R) will be observed and a field in the opposite direction will be required to reduce the magnetization to zero. This point is called coercivity field (H_C). Therefore, in the case of multi-domain NPs an open hysteresis loop, with a non-negligible inner area, can be observed. In the case of superparamagnetic NPs, as the net magnetization is zero when the magnetic field is zero, the

coercivity is zero and the inner area of the hysteresis loop is negligible. b, Schematic representation of the temperature dependence of the AC magnetic susceptibility related to MNP aggregation status. χ'' = imaginary part. Only one type of MNP was used to prepare the nHs, with a concrete composition and size distribution. Thus, the main aspect affecting the temperature maxima location of the in-phase and out-of-phase susceptibility AC measurements are the interparticle interactions, indicating a higher degree of aggregation of the particles when the maxima is shifted towards higher temperatures.

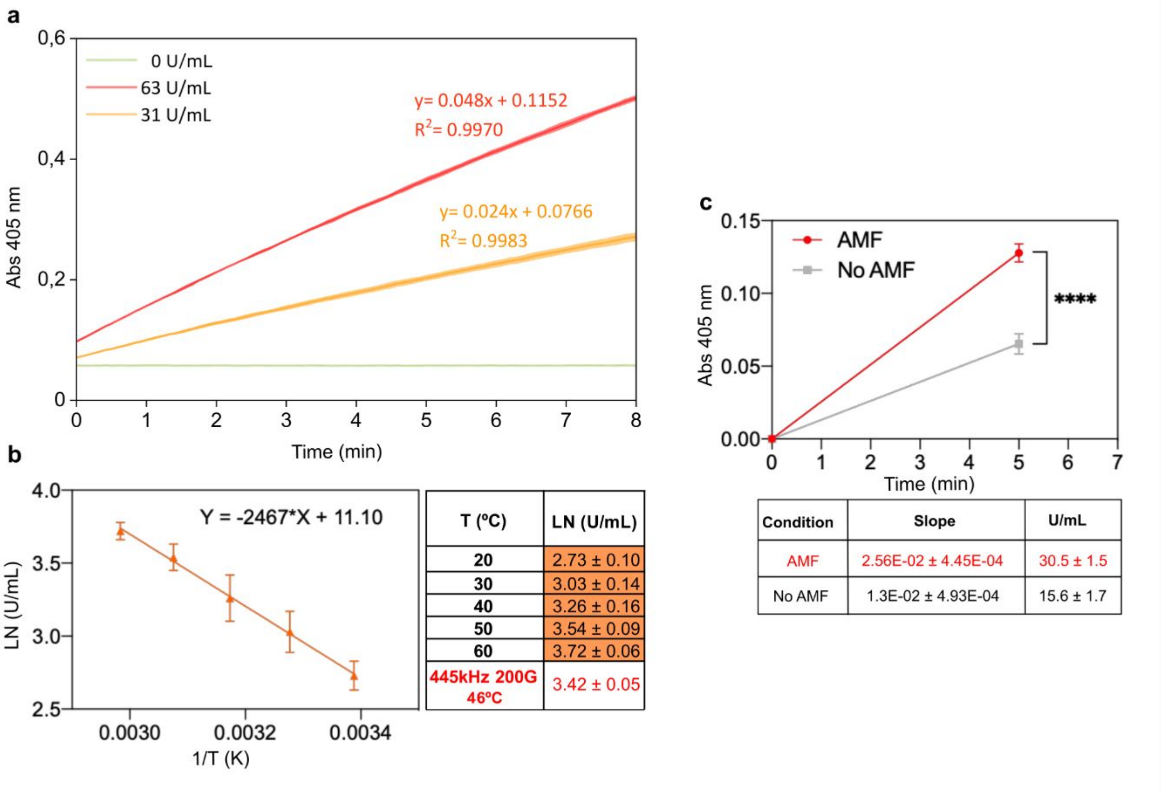

T (ºC)	LN (U/mL)
20	2.73 ± 0.10
30	3.03 ± 0.14
40	3.26 ± 0.16
50	3.54 ± 0.09
60	3.72 ± 0.06
445kHz 200G 46ºC	3.42 ± 0.05

Condition	Slope	U/mL
AMF	2.56E-02 ± 4.45E-04	30.5 ± 1.5
No AMF	1.3E-02 ± 4.93E-04	15.6 ± 1.7

Figure S8. Inferring T_{LOC} in nHs upon AMF application from Arrhenius linearization plot. a, Linearity of the colorimetric assay used to determine the initial rate of ABTS oxidation of HRP nHs measuring the increase of 405 nm with time. *Activity assay:* 1.7 mL of 0.1M potassium phosphate, pH 5.0 at 25 *˚C*, 0.1 mL of 9.1 mM ABTS, 0.2 mL 0.3 % (w/w)

hydrogen peroxide solution (H_2O_2) in deionized water. b, Arrhenius linearization plot including the LN (U/mL) values obtained for initial ABTS oxidation rates when nHs were heated globally at different temperatures using a thermoblock. The main LN (U/mL) value obtained upon AMF application (n=8) is also included. c, Difference in initial ABTS oxidation rate upon exposure (red line) or not (gray line) of the reaction mixture to AMF (*f* of 445 kHz and *H* of 200 G). Endpoint assay was performed within the linearity time scale of the enzyme assay using the same reaction conditions and total protein concentration in the cuvette either when AMF was or was not applied. Mean ± s.d, n= 8. Unpaired T-Test (****p <0.0001).

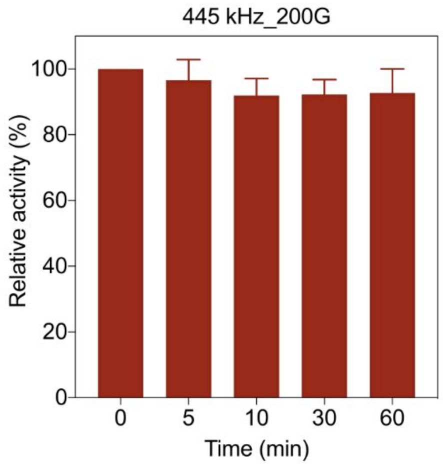

Figure S9. AMF stability of nHs concerning the application of an AMF of 445 kHz and 20mT. nHs were incubated in 0.1M sodium phosphate pH 8.0 buffer during AMF application**.** Histogram represents the remaining activity of the entrapped HRP measured after being exposed to this AMF condition for different periods. Mean ± s.d, n= 3. Unpaired T-Test.

a

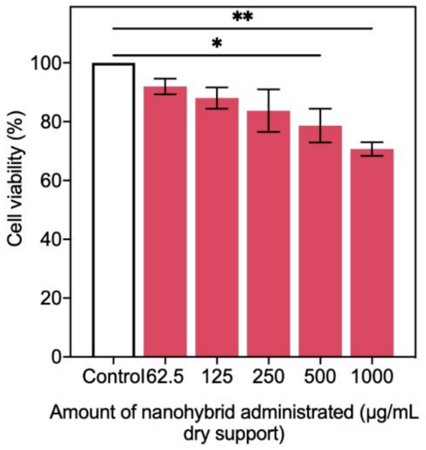

b

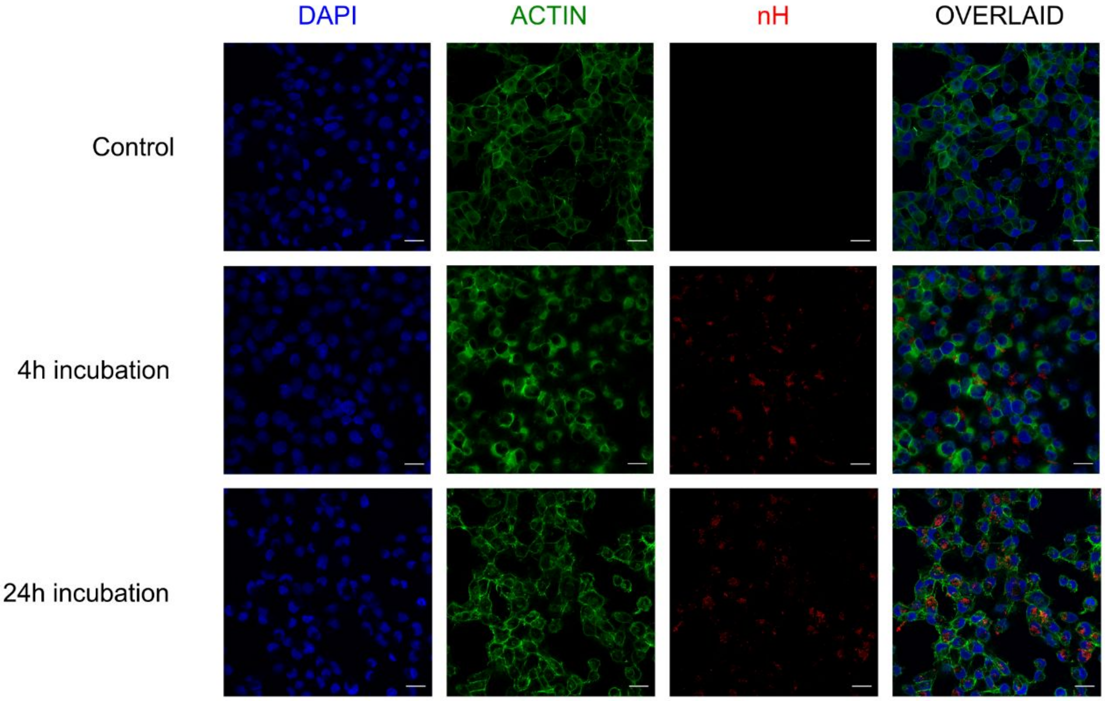

Figure S10. Effect of the obtained nHs at the cellular level using MIA PaCa-2 human pancreatic carcinoma as cell line. a, Cytotoxicity of nHs. Their intrinsic cytotoxicity was measured by MTT after 24h of incubation. Histogram represents mean ± S.E.M, n= 3.

Unpaired T-Test (*$p<0.05$, **$p<0.01$). b, nHs interaction with cells analyzed by confocal microscopy. MIA PaCa-2 cells were incubated with the nHs (200 µg mL^{-1}) for 4h or 24 h. Nuclei were stained with DAPI (blue), and actin filaments were stained with Phalloidin-488 (green). nHs were detected by iron self-reflection signal (artificially colored in red). Scale bar: 20 µm.

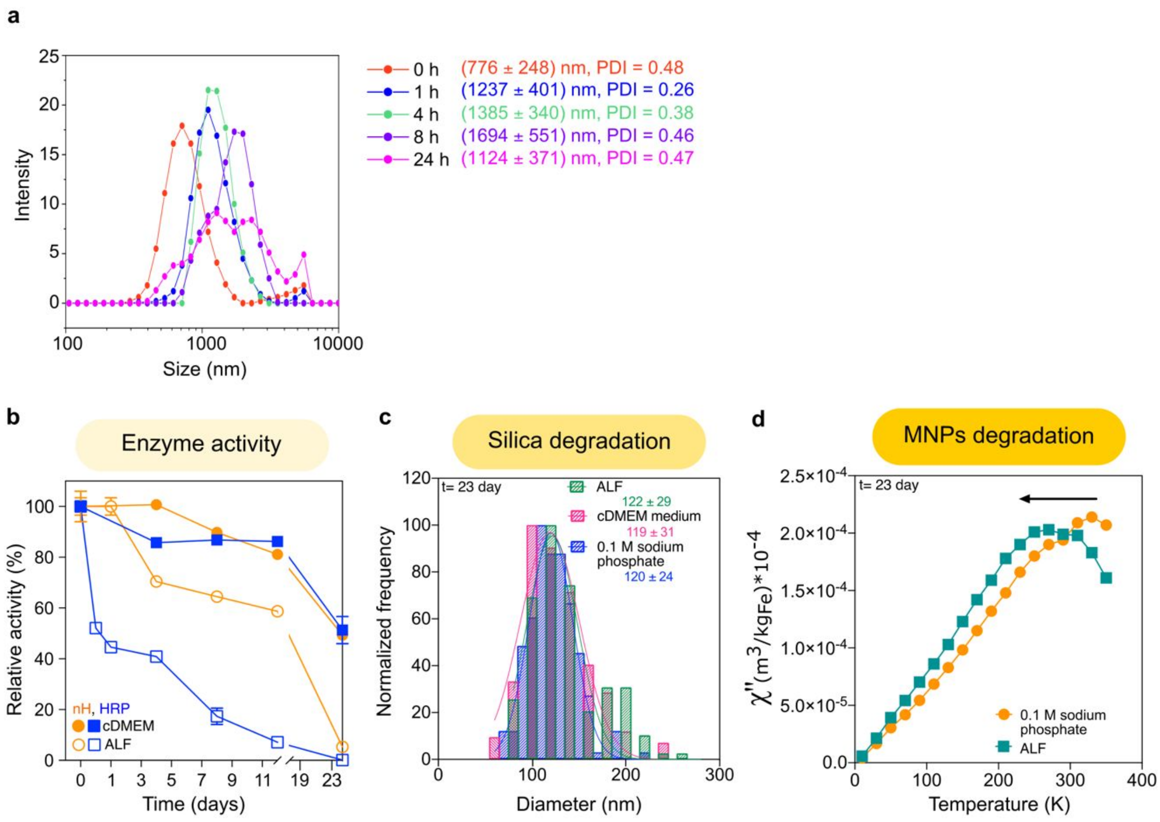

Figure S11. nH stability in complex media. a, Stability of nHs incubated in DMEM supplemented with 10 % FBS over time. The hydrodynamic size of nHs at different time points (0, 1, 4, 8, and 24) was determined by DLS. The hydrodynamic diameter and polydispersity indices (PDI) are shown in all cases. b, Stability of the enzymatic activity of soluble (blue square) and entrapped (orange circle) HRP. The relative activity of the HRP in cDMEM (colored) and artificial lysosomal fluid (ALF) (clear) are depicted. Half-lives (t ½) were calculated from one-phase exponential decay functions which were fitted to individual stability points in GraphPad. The t ½ for the soluble enzyme was 0.04 days whereas t ½ for the nHs was 9.96 which is ~249 stabilization factor c, Histogram and log-normal fitting curves of the size distribution of nHs incubated in different media for 23 days: ALF pH 5.0 (green), cDMEM pH 7.4 (pink) and 0.1 M sodium phosphate pH 8.0 (blue). The size of the

different preparations was measured by TEM. d, Temperature dependence of the AC magnetic susceptibility (out-of-phase component) of nHs incubated in phosphate buffer (orange circle) and nHs incubated in ALF (dark green square) after 23 days.

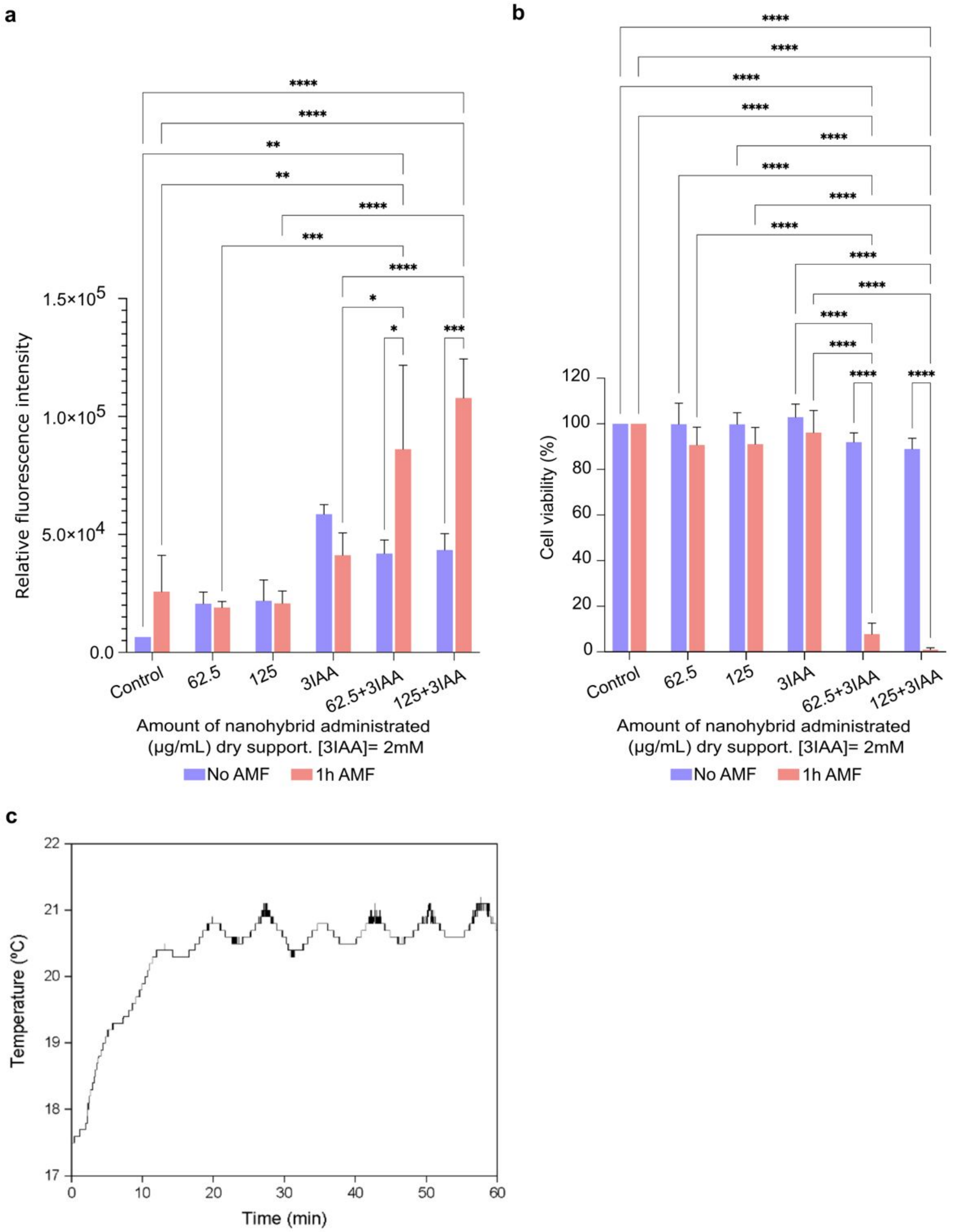

Figure S12. a, Additional controls performed to evaluate ROS production. b, Additional controls were performed to evaluate cytotoxicity by MTT triggered by enzyme prodrug AMF-mediated nHs. The controls were performed under the same experimental conditions as the assays described in Figure 4d,e. Cell samples to which no nHs were added, were used as control of a possible direct effect of applying AMF to cells (Control 1). Cell samples to which 125 μg mL^{-1} or 62.5 of nHs were added, but not the prodrug, were used to assess a possible direct effect of the magnetic heating triggered by the entrapped MNPs within nHs when AMF was applied (Control 2 and Control 3, respectively). ROS production and the biological response were determined by whether AMF was applied (red bars) or not (purple bars). Histograms represent mean ± S.E.M, n = 3. Two-way ANOVA, followed by Tukey's multiple comparisons test. c, Representative measurement of the global temperature variation of the culture medium when applying AMF. Measurements were performed continuously using an optical fiber thermal probe in all cell culture samples on which the proposed therapy was applied

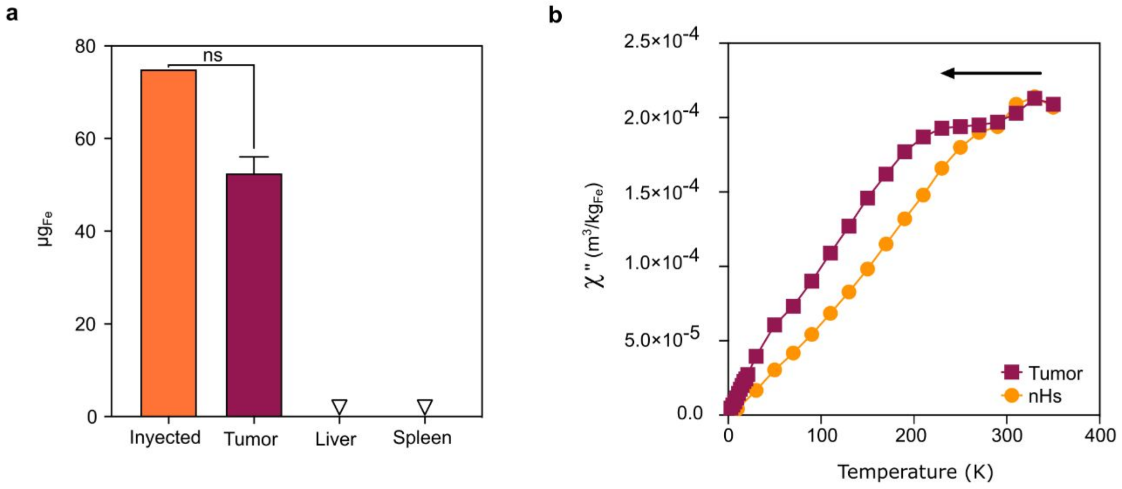

Figure S13. a, Quantification of the nanoparticles that accumulate in the tumor, liver, and spleen after 23 days. The AC magnetic susceptibility curves of the tumor, spleen, and liver

were compared with the known injected amount of nHs. The majority of the injected nHs are presented in the tumor, while the amount of nHs in the liver and spleen is below the limit of detection. Histogram represents mean ± sd, (n = 3 for liver and spleen, n=2 for tumor). Unpaired T-Test. b, Analysis of the metabolization of the nHs after 23 days in the tumor measured by AC magnetic susceptibility.

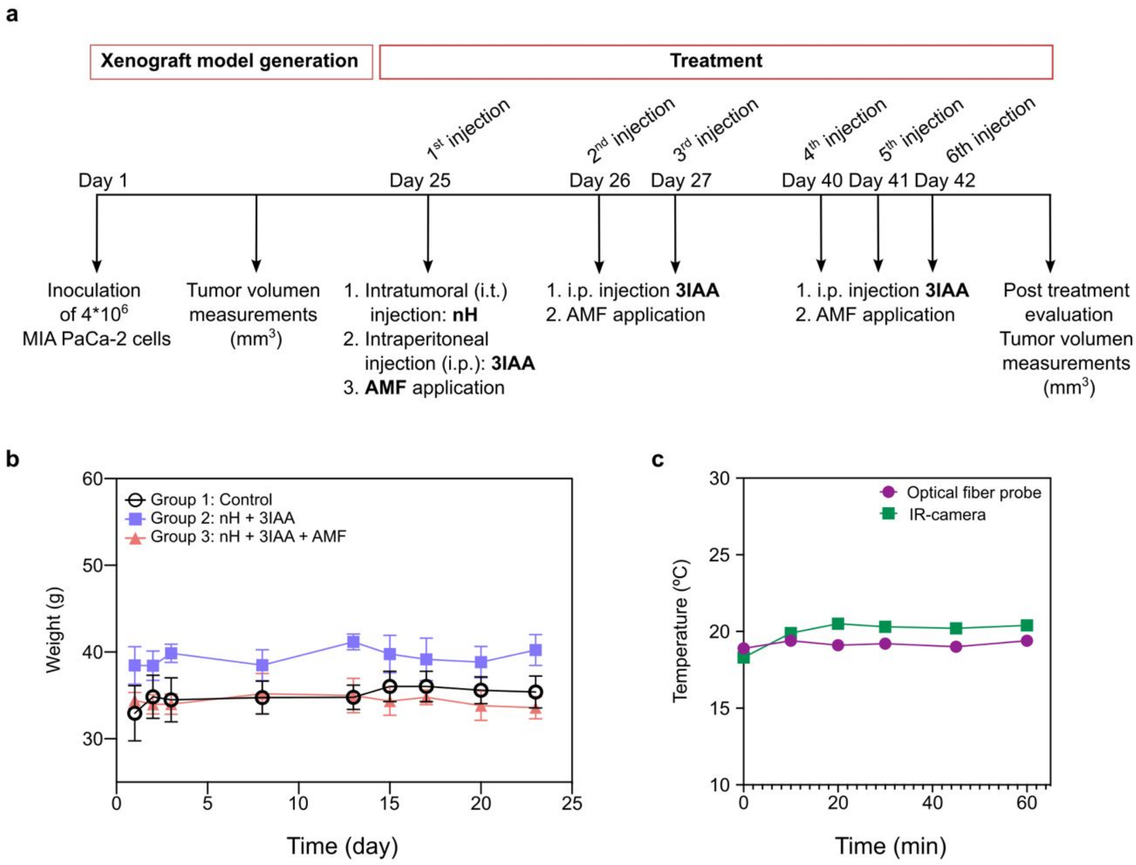

Figure S14. a, Workflow followed for the generation of MIA Paca-2 tumor xenografts and subsequent enzyme-prodrug treatment. b, Comparison of body weight changes of the different mice groups during treatment. Data are shown as mean ± standard deviation (n = 4 per group). c, *Ex vivo* global temperature measurements over time upon application of AMF. AMF conditions and nHs concentrations were the same as in *in vivo* experiments.

Table S1: Summary of the main characteristics of FluidMAG-PAS, including particle-size distribution determined by TEM and DLS, and Z-potential.

MNPs	Core	Coating	Hydrodynamic diameter (nm)	PDI	Diameter (TEM) (nm)	Z-potential (mV)
FluidMAG-PAS	Fe_2O_3	Polyacrilic acid	203 (Intensity) 193 (Number)	0.092 ± 0.02	12.34 ± 1.8	-19 ± 4

Table S2. Studies of the conditions of silica deposition and co-encapsulation for the best specific activity of the obtained nHs. _Condition 3_ is highlighted in green as it turned out the optimal condition and the one that was, therefore, selected for further experimentation.

Condition 1	Volume (mL)	Final Molarity (mM)	Final µmol	Final Equivalents
Phosphate ions	0.40	68	40	10
PEI MW 1300	0.05	7	4.20	1
TMOS	0.10	170	100	24
HRP	0.04	0.02	9.00E-04	2.14E-04

Condition 2	Volume (mL)	Final Molarity (mM)	Final µmol	Final Equivalents
Phosphate ions	0.40	68	40	482
PEI MW 60000	0.05	0.14	0.08	1
TMOS	0.10	170	100	1205
HRP	0.04	0.02	9.00E-04	0.01

**Condition 3**	Volume (mL)	Final Molarity (mM)	Final µmol	Final Equivalents
Phosphate ions	0.40	3	2	0.5
PEI MW 1300	0.05	7.00	4.20	1
TMOS	0.10	170	100	24
HRP	0.04	0.02	9.00E-04	2.14E-04

Condition 4	Volume (mL)	Final Molarity (mM)	Final µmol	Final Equivalents
Phosphate ions	0.40	3.4	2	24
PEI MW 60000	0.05	0.14	0.08	1

TMOS	0.10	170	100	1205
HRP	0.04	0.02	9.00E-04	0.01

Table S3. Effects of the different tested conditions according to Table S1. The entrapment efficiency parameters and SEM images of the silica nanoparticles are shown. Scale bar = 1 μm. Condition 3 was selected according to the activity and the size of the synthesized particles.

	Enzyme immobilization (%)	Expressed activity (%)	U/mL	Entrapped iron (%)	SEM micrograph
**Condition 1**	87	57	26	69	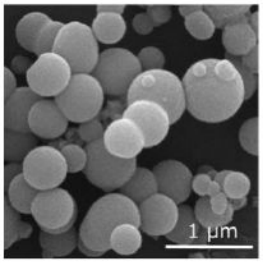
**Condition 2**	75	34	13	61	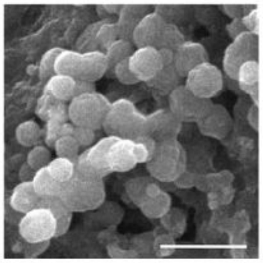
**Condition 3**	68	60	21	90	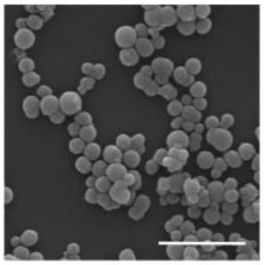
**Condition 4**	34	3	0	ND	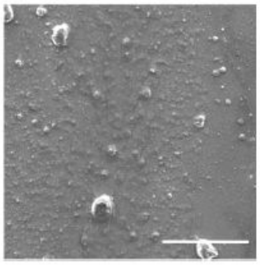

Table S4. Entrapment parameters for nHs synthesis. a**,** Enzyme and iron entrapment efficiency parameters of four synthesis replicates using Condition 3 that was selected as optimal. Equations for enzyme immobilization and expressed activity calculation are shown. b, The calculations for determining the number of MNPs entrapped /mg silica calculations are explained). c, Table summarizing the average entrapment efficiency of the nHs replicates and a comparison with the soluble enzyme specific activity. Data are shown as mean ± sd (n=4).

a) Co-entrapment replicates

Synthesis replicates	Sample	U/mL	Enzyme immobilization (%)	Expressed activity (%)	Specific activity (U/mg enzyme)	Support concentration (mg/mL)	Specific activity (U/mg dry support)	Entrapped iron (mg/mL)	*n° of MNPs entrapped /mg silica
1	HRP soluble	45.00			450.00				
	nHs	22.00	71.00	70.00	309.86	34.00	0.65	0.80	5.58E+12
2	HRP soluble	48.00			480.00				
	nHs	21.00	70.83	59.20	296.48	44.00	0.48	0.80	4.31E+12
3	HRP soluble	57.00			570.00				
	nHs	20.00	73.00	53.00	273.97	42.00	0.48	1.00	5.65E+12
4	HRP soluble	53.00			530.00				
	nHs	20.00	66.00	58.00	303.03	31.00	0.65	0.97	7.43E+12

$$\% I = \frac{Initial\ activity - Activity\ in\ supernatant}{Initial\ activity} * 100$$

$$\% EA = \frac{Activity\ in\ immobilized\ preparation}{Initial\ activity - Activity\ in\ supernatant} * 100$$

b) n° of MNPs entrapped /mg silica calculations:

	**Density (g/cm³)**
**Fe_2O_3**	5.24
**Fe**	7.87

Synthesis	Shape	diameter (nm)	Radius NP (nm)	Volume NP (cm³)	Area/surface area NP (nm²)	weight NP (mg)	n° NPs/mg Fe_2O_3	Entrapped iron mg/mL	Fe_2O_3 weight (mg)	n° of MNPs entrapped	n° of MNPs entrapped /mg silica
1	spheric	13	6.5	1.15E-18	530.66	6.02E-15	1.66E+14	0.8	0.46	7.59E+13	5.58E+12
2	spheric	13	6.5	1.15E-18	530.66	6.02E-15	1.66E+14	0.8	0.46	7.59E+13	4.31E+12
3	spheric	13	6.5	1.15E-18	530.66	6.02E-15	1.66E+14	1	0.57	9.49E+13	5.65E+12
4	spheric	13	6.5	1.15E-18	530.66	6.02E-15	1.66E+14	0.97	0.55	9.21E+13	7.43E+12

c) nHs average entrapment efficiency, and entrapped vs soluble specific activity

	**Enzyme immobilization (%)**	**Expressed activity (%)**	**Specific activity (U/mg enzyme)**	**Specific activity (U/mg dry support)**	**Entrapped iron mg/mL**	**n° of MNPs entrapped /mg silica**
**nHs**	68 ± 3	60 ± 7	296 ± 15	0.56 ± 0.1	0.9 ± 0.1	5.74E+12 ± 1.27E+12
**HRP soluble**			507 ± 53			

Table S5. Determination of endotoxin presence in nHs

	OD 405 nm		Blank subtraction		EU/ml		EU/ml average
	measure 1	measure 2	measure 1	measure 2	measure 1	measure 2	
0.1	0.79	0.77	0.43	0.40	0.11	0.10	0.10
0.05	0.62	0.62	0.25	0.25	0.06	0.06	0.06
0.025	0.49	0.49	0.12	0.12	0.03	0.03	0.03
0.01	0.42	0.43	0.05	0.07	0.01	0.02	0.01
Blank	0.37	0.36	0.00	0.00	0.00	0.00	0.00

Standard curve	
EU/ml	OD (405 nm) - blank
0.1	0.41
0.05	0.25
0.025	0.12
0.01	0.06
0	0

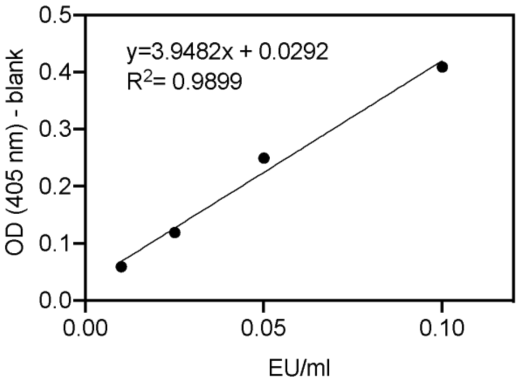

ug/ml nH	Spiked endotox	OD 405 nm		EU/ml		EU/ml average	Endotox contamination
		measure 1	measure 2	measure 1	measure 2		
100	No	0.21	0.23	-0.04	-0.04	-0.04	No
100	Yes	0.50	0.44	0.03	0.02	0.03	
10	No	0.38	0.37	0.00	0.00	0.00	No
10	Yes	0.42	0.64	0.01	0.07	0.04	

REFERENCES

(1) Ouellette, R. J.; Rawn, J. D. Principles of Organic Chemistry; Elsevier: Amsterdam ; Boston, 2015.

(2) Tomasello, G.; Armenia, I.; Molla, G. The Protein Imager: A Full-Featured Online Molecular Viewer Interface with Server-Side HQ-Rendering Capabilities. Bioinformatics 2020, 36 (9), 2909–2911. https://doi.org/10.1093/bioinformatics/btaa009.
